# Comprehensive Genome-Wide Identification and Expression Profiling of *Eceriferum* (*CER*) Gene Family in Passion Fruit (*Passiflora edulis*) Under *Fusarium kyushuense* and Drought Stress Conditions

**DOI:** 10.3389/fpls.2022.898307

**Published:** 2022-06-27

**Authors:** Hafiz Muhammad Rizwan, Abdul Waheed, Songfeng Ma, Jiankun Li, Muhammad Bilal Arshad, Muhammad Irshad, Binqi Li, Xuelian Yang, Ahmad Ali, Mohamed A. A. Ahmed, Nusrat Shaheen, Sandra S. Scholz, Ralf Oelmüller, Zhimin Lin, Faxing Chen

**Affiliations:** ^1^College of Horticulture, Fujian Agriculture and Forestry University, Fuzhou, China; ^2^Key Laboratory for Bio Pesticide and Chemical Biology, Ministry of Education, Fujian Agriculture and Forestry University, Fuzhou, China; ^3^Department of Plant Breeding and Genetics, College of Agriculture, University of Sargodha, Sargodha, Pakistan; ^4^College of Horticulture, The University of Agriculture, Peshawar, Pakistan; ^5^National Engineering Research Center for Sugarcane, Fujian Agriculture and Forestry University, Fuzhou, China; ^6^Plant Production Department (Horticulture-Medicinal and Aromatic Plants), Faculty of Agriculture (Saba Basha), Alexandria University, Alexandria, Egypt; ^7^Department of Chemistry, Abbottabad University of Science and Technology, Abbottabad, Pakistan; ^8^Matthias Schleiden Institute, Plant Physiology, Friedrich-Schiller-University Jena, Jena, Germany; ^9^Institute of Biotechnology, Fujian Academy of Agricultural Sciences, Fuzhou, China

**Keywords:** cuticle wax biosynthesis, synteny, gene ontology, micro-RNA, stress conditions

## Abstract

Plant surfaces are covered with cuticle wax and are the first barrier between a plant and environmental stresses. *Eceriferum* (*CER*) is an important gene family involved in wax biosynthesis and stress resistance. In this study, for the first time, 34 *CER* genes were identified in the passion fruit (*Passiflora edulis*) genome, and PeCER proteins varied in physicochemical properties. A phylogenetic tree was constructed and divided into seven clades to identify the evolutionary relationship with other plant species. Gene structure analyses revealed that conserved motifs ranged from 1 to 24, and that exons ranged from 1 to 29. The *cis*-element analysis provides insight into possible roles of *PeCER* genes in plant growth, development and stress responses. The syntenic analysis revealed that segmental (six gene pairs) and tandem (six gene pairs) gene duplication played an important role in the expansion of *PeCER* genes and underwent a strong purifying selection. In addition, 12 putative ped-miRNAs were identified to be targeting 16 *PeCER* genes, and *PeCER6* was the most targeted by four miRNAs including ped-miR157a-5p, ped-miR164b-5p, ped-miR319b, and ped-miR319l. Potential transcription factors (TFs) such as ERF, AP2, MYB, and bZIP were predicted and visualized in a TF regulatory network interacting with *PeCER* genes. GO and KEGG annotation analysis revealed that *PeCER* genes were highly related to fatty acid, cutin, and wax biosynthesis, plant-pathogen interactions, and stress response pathways. The hypothesis that most PeCER proteins were predicted to localize to the plasma membrane was validated by transient expression assays of PeCER32 protein in onion epidermal cells. qRT-PCR expression results showed that most of the *PeCER* genes including *PeCER1, PeCER11, PeCER15, PeCER17*, and *PeCER32* were upregulated under drought and *Fusarium kyushuense* stress conditions compared to controls. These findings provide a foundation for further studies on functions of *PeCER* genes to further facilitate the genetic modification of passion fruit wax biosynthesis and stress resistance.

## Introduction

Plant growth and development are significantly affected by different environmental factors such as biotic and abiotic stresses (Pandey et al., 2017; Raza, 2021; Sharif et al., 2021). The plant cuticle is a hydrophobic protective layer covering plant epidermis and is the first barrier between the environment and plants. Epicuticle wax is composed of very-long-chain fatty acids (VLCFAs) and their derivatives such as alkanes, ketones, primary and secondary alcohols, aldehydes, esters, and triterpenes (Zhang et al., 2020). The wax of plant cuticles play an important role in controlling non-stomatal water loss and preventing mechanical damages, ultraviolet (UV) light, pathogens, and other environmental biotic and abiotic stresses (Lewandowska et al., 2020). Plant leaves without wax tend to have high transpiration rate, low chlorophyll content, and inferior CO_2_ assimilation ability (Medeiros et al., 2017). According to reports, fruit quality and shelf life are also associated with the development and structure of wax on fruit surfaces (Lara et al., 2019; Ding et al., 2020; Zhang et al., 2021).

*Eceriferum* (*CER*) genes play an important role in VLCFAs and wax biosynthesis pathways, and have been thoroughly investigated in numerous plant species (Li et al., 2021). The first member, the *CER1* gene, was molecularly characterized in *Arabidopsis* (*AtCER1*) and was discovered to be responsible for the conversion of ultra-long-chain aldehydes into ultra-long-chain alkanes and stimulated under biotic and abiotic stresses (Aarts et al., 1995; Wang et al., 2021). The overexpression of *OsCER1* in rice (*Oryza sativa*) involved the biosynthesis of VLCFAs and alkanes, differentiation of plastids, and pollen development (Ni et al., 2018). Wang et al. (2020) also pointed out that *BnCER1-2* in overexpressed rapeseed (*Brassica napus*) plants stimulated the production of alkanes and improved the tolerance to drought. Sakuradani et al. (2013) found that the *CER22* gene, an allele of *CER1*, was involved in the biosynthesis of cuticular wax alkanes in *Arabidopsis*. *AtCER2* was found to be involved in VLCFA biosynthesis and acyltransferase in C_28_ elongation process (Pascal et al., 2013) as well as in pollen coat and cuticle formation (Haslam et al., 2015). *CER3* was found to be involved in epidermal wax biosynthesis in response to changes in humidity in *Arabidopsis. CER3* interacts with *CER1* and catalyzes redox-dependent VLCFAs from very long chain acyl-CoA (Rowland et al., 2007; Kim et al., 2019). *CER4* encodes fatty acyl-CoA that forms alcohol reductase and is involved in the ester reduction pathway for production of *Arabidopsis* epidermal wax. *CER4* has been reported to be expressed in different plant tissues including plant stem, roots, flowers, leaves, and siliques (Rowland et al., 2006). Adenosine triphosphate-binding cassette (ABC) proteins are transporters in endogenous substrates across the plasma and intracellular membranes (Sharom et al., 2011). Epicuticular wax is produced in epidermal cells and transported to the cuticle *via* ABC transporters. Pighin et al. (2004) reported that the *Arabidopsis CER5* gene encodes an ABC transporter, which is required for wax export to the cuticle and localized in the plasma membrane of epidermal cells. The overexpression of the *CER6* gene in *Arabidopsis* showed a significant increase in the production of stem wax (Hooker et al., 2002), as well as in the production of fatty acids from C_26_ to C_28_ (Tresch et al., 2012).

*Arabidopsis CER7* mutants participate in wax biosynthesis by reducing the expression level of *CER3/WAX2* and promoting cellular procedures (Hooker et al., 2007). Lü et al. (2009) reported that the *Arabidopsis CER8/LACS* (long-chain acyl-CoA-synthetase-1) mutant showed an overlapping function with *LACS2* in cuticle wax biosynthesis. *CER9* was involved in cuticle biosynthesis and maintained plant water level by encoding the putative E3 ubiquitin ligase in *Arabidopsis* and participating in the signal transduction of abscisic acid (ABA) in seeds and seedlings (Zhao et al., 2014). Zheng et al. (2005) reported that *CER10* was involved in VLCFA biosynthesis, and later on, Ensing et al. (2016) further identified that the *CER10* mutant exhibited an increase in non-stomatal water loss and was tolerant to drought conditions. Pascal et al. (2013) demonstrated that the CER26 and CER2-like proteins were involved in the elongation of VLCFAs and wax biosynthesis. The overexpression of *CER26* mutants exhibited a glossy stem phenotype and C_30_ carbon leading to cuticle wax biosynthesis. *CER60* was involved in VLCFA, which prolonged C_28_ to C_30_ activity and produces trace amounts of VLCFA after expressed in yeast (Hooker et al., 2002; Trenkamp et al., 2004).

Cuticular wax plays an important role in plant biotic and abiotic stresses. Drought is the main type of abiotic stress, limiting the growth and productivity of plants and leading to global food security and terrestrial health (Kumar et al., 2021). The expression levels of *CER* genes were upregulated in response to drought, osmotic, and ABA responses (Xue et al., 2017). The ectopic overexpression of *AtCER1* and *AtCER3* genes in tobacco improved the cuticle wax and reduced water loss under drought stress conditions (Cameron et al., 2006). Wang et al. (2015) reported that the overexpression of cucumber (*Cucumis sativus*) *CsWAX2* homologous to *CER3* increased resistance to drought and pathogens. The biosynthesis of cuticle wax is mediated by different transcription factors (TFs) and the coordinated expression of multiple related genes (Al-Abdallat et al., 2014). Different TFs have been reported to be involved in the biosynthesis of fatty acids and wax including AP2/ERF (APETALA2/ETHYLENE RESPONSIVE FACTOR), WIN1 (WAX INDUCER1), SHN1 (SHINE1), and WRI1 (WRINKLED1) (Hao et al., 2017).

In addition, micro-RNAs (miRNAs) are non-coding single-stranded RNAs (~21–30 nucleotides) found in plants and animals. It has been reported that miRNAs play an important role in numerous cellular mechanisms as well as in stress resistance by translational inhibition and/or cleavage of target mRNAs during or after transcription (Yadav et al., 2020). The miRNA, such as trans-acting small interfering RNA (tasiRNA) in *Arabidopsis thaliana* has been reported to be involved in *CER3* silencing during stem wax production (Lam et al., 2015). Similarly, Liu et al. (2019) mentioned that brassica miRNA (bna-miR165a-5p) may be involved in the synthesis of wax by regulating the *BnaA06g40560D* gene. These findings suggested that miRNA might have a significant role in wax biosynthesis by modulating targeted genes. Gene Ontology (GO) is a collection of ideas described in terms that aimed to classify genes on the basis of function and consists of three main classifications, biological processes (GO-BP), cellular component (GO-CC), and molecular function (GO-MF). Using advanced GO terms, functional classification of genes can be conducted on species (Ashburner et al., 2000; Raza et al., 2022). The Kyoto Encyclopedia of Genes and Genomes (KEGG) is a bioinformatics resource for understanding biological functions at genomic levels, and contains network information to present functions in pathways (Masoudi-Nejad et al., 2007).

The *CER* gene family has been identified in different plant species including 29 *CER* family members in jujuba (*Ziziphus jujuba* Mill.) (Li et al., 2021), 10 *CER* family members in apple (*Malus domestica*) (Qi et al., 2019a), and 37 *CER* family members in sunflower (*Helianthus annuus*) (Ahmad et al., 2021). However, the *CER* gene family has not been identified in passion fruit (*Passiflora edulis*). Passion fruit is a perennial climbing vine and is usually cultivated throughout tropical and subtropical regions of the world. Passion fruit is an economically important fruit plant and is well-known for its fresh juice, rich aroma, and distinctive nutritional values (Rizwan et al., 2021). Recently, the passion fruit genome (Ma et al., 2021) has been published. For the first time, a comprehensive genome-wide study was conducted to identify the *CER* genes in the passion fruit genome. Moreover, their *in-silico* prediction of TFs, subcellular localization, evolutionary relationship *via* phylogenic and syntenic analysis, conserved motifs, gene structures, *cis*-regulatory elements, prediction of putative miRNA, and functional annotation analyses were performed. qRT-PCR expression profiles in different passion fruit tissues under drought stress (abiotic stress) and *Fusarium kyushuense* fungal stress (biotic stress) conditions were identified to gain insight into passion fruit *CER* genes. This study provides a foundation for further functional studies on candidate passion fruit *CER* genes to further genetic improvement of passion fruit.

## Materials and Methods

### Passion Fruit CER Gene Identification

In order to perform the genome-wide identification of *CER* genes in passion fruit, all known CER family protein sequences of *Arabidopsis thaliana* (AtCER) were downloaded from The *Arabidopsis* Information Resource (TAIR) database (http://www.Arabidopsis.org), *Malus domestica* (apple, MdCER) (Qi et al., 2019a) from the Phytozome database (https://data.jgi.doe.gov/refine-download/phytozome?organism=Mdomestica&expanded=491), and *Helianthus annuus* (sunflower, HaCER) (Ahmad et al., 2021) and *Ziziphus jujuba* (jujuba, ZjCER) (Li et al., 2021) from National Center for Biotechnology Information (NCBI) (https://www.ncbi.nlm.nih.gov/). The passion fruit protein, CDS (coding DNA sequence), gff, and genome sequence files were downloaded from the passion fruit genome (http://ftp.cngb.org/pub/CNSA/data3/CNP0001287/CNS0275691/CNA0017758/) (Ma et al., 2021). To identify the homologous *CER* genes in the passion fruit genome, BLASTp (Basic Local Alignment Search Tool for proteins) was performed in default mode using the protein sequences in TBtools software package v 1.098726 (https://github.com/CJ-Chen/TBtools/releases/tag/1.098726) (Chen C. et al., 2020).

### Physicochemical Features and Phylogenetic Analyses of *PeCER* Genes

Physicochemical features such as molecular weight (MW), CDS length, amino acid (AA) length, instability index (II), aliphatic index (AI), isoelectric point (pl), number of exons and introns (E/I) of *PeCER* genes were evaluated using the ExPASY-Prot online tool (https://web.expasy.org/protparam/) (Gasteiger et al., 2005). Subcellular localization of *PeCER* genes was predicted using CELLO v.2.5 (http://cello.life.nctu.edu.tw) (Yu et al., 2004). Full-length protein sequences of the *CER* genes of *P. edulis, A. thaliana, M. domestica, H. annus*, and *Z. jujuba* were aligned with Molecular Evolutionary Genetics Analyses (MEGA) software v 10.1.8 (https://www.megasoftware.net/) (Kumar et al., 2008) and were used for phylogenetic analyses. A neighbor-joining (NJ) tree was constructed using the MEGA software with 1,000 bootstrap replicates, and all other parameters were set to default. The iTOL web tool (https://itol.embl.de/login.cgi) (Letunic and Bork, 2007) was used to visualize the phylogenetic tree and divide it into clades. Passion fruit genomic files were used to investigate the distribution of *PeCER* genes on all passion fruit chromosomes with the TBtools software (Chen C. et al., 2020). Moreover, the online SIAS (Sequence Identity and Similarity) (http://imed.med.ucm.es/Tools/sias.html) tool was used to perform pairwise sequence identification on passion fruit *CER* genes.

### Determination of *PeCER* Gene Structures and Motifs Analyses

Gene structure annotations for candidate *CER* genes were retrieved from passion fruit gff genomic files. Conserved motifs of PeCER proteins were recognized by employing the MEME (Multiple Expectation Maximization for Motif Elicitation) online tool (http://meme-suite.org/index.html) (Bailey et al., 2006), and the number of motifs was set to fifteen. The TBtools software was used to merge and visualize *PeCER* gene structures and conserved motifs from passion fruit.

### *PeCER* Gene *cis*-Regulatory Element Analysis

Two-thousand-bp sequences of upstream regions of all *PeCER* genes were extracted from genomic DNA sequences for the prediction of putative *cis*-regulatory elements. Furthermore, the extracted sequences (2,000 bp) were subjected to the online PlantCARE database (https://bioinformatics.psb.ugent.be/ webtools/plantcare/html/) (Lescot, 2002), and a figure was drawn using the TBtools software (Chen C. et al., 2020). The numbers, functions, and sequences of putative *cis*-acting elements of *PeCER* genes were summarized and highlighted into different categories including plant growth and development, phytohormone responses, and stress responses.

### *PeCER* Gene Synteny Analysis and Calculation of Ka/Ks Values

Segmental and tandem duplication provides new insight into family gene development and genome progression. Homologous *PeCER* genes on the same passion fruit chromosome with no more than one intervention gene were considered tandem duplicates, while on other chromosomes were considered segmental duplicates. *PeCER* gene duplicates were identified and visualized according to their physical position on chromosomes in the passion fruit genome. Gene duplication, synteny, and non-synonymous (Ka)/synonymous (Ks) analysis of *CER* genes were performed with the TBtools software (Chen C. et al., 2020). The syntenic relationships of CER genes between *P. edulis, A. thaliana, M. domestica, H. annus*, and *Z. jujuba* were performed by TBtools software using MCScanX toolkit package (Chen C. et al., 2020). Ka and Ks nucleotide substitution rates and Ka/Ks ratios were annotated using TBtools, and divergence time (mya = millions year ago) was calculated as follows: time = Ks/2x (x = 6.38 × 10^−9^) (Ma et al., 2021).

### PeCER Protein-Protein Interaction, Secondary Structure Prediction, and 3D Modeling

The PeCER protein-protein interaction network was predicted using the online tool STRING 11.0 (https://string-db.org/cgi/input.pl), and the interaction was constructed based on known *Arabidopsis* proteins. The parameters for the STRING tool were set as follows: network type-full STRING network: the meaning of network edge evidence; minimum required interaction score was set to medium confidence parameter (0.4), and the maximum number of interactors to show was no more than 10. The protein secondary structures of PeCER were predicted using SOPMA SECONDARY STRUCTURE PREDICTION METHOD (https://npsa-prabi.ibcp.fr/cgi-bin/npsa_automat.pl?page=npsa_sopma.html) (Geourjon and Deleage, 1995). All 34 PeCER proteins were three-dimensionally modeled (3D) using Phyre2 with default mode (http://www.sbg.bio.ic.ac.uk/phyre2/html/) (Kelley et al., 2015).

### Putative miRNAs Targeting *PeCER* Gene Prediction and GO and KEGG Annotation Analyses

The potential miRNA target sites in *PeCER* genes were predicted by the following steps; first, the published mature sequences of passion fruit miRNAs were downloaded (Paul et al., 2020), later the CDS sequences of all the 34 PeCER genes were submitted to the psRNATarget server with default parameters (https://www.zhaolab.org/psRNATarget/) for the prediction of potential miRNAs in PeCER genes. Cytoscape software V 3.9 (https://cytoscape.org/download.html) was used to visualize the interaction network between the predicted miRNAs and *PeCER* target genes. The GO and KEGG annotation analyses were performed by submitting all the 34 PeCER protein sequences to the online database eggNOG (http://eggnog-mapper.embl.de/), and their enrichment and annotation analysis were performed with the TBtools software (Chen C. et al., 2020).

### Plant Transcription Factor Regulatory Network Analysis of *PeCER* Genes

The plant TF prediction and regulatory network analysis were performed as described by Zheng et al. (2020) with slight modifications. In order to predict the TFs in the upstream regions of PeCER genes, 1,000-bp nucleotide sequences from the upstream promoter region of *PeCER* genes were extracted from the passion fruit genome and submitted to Plant Transcriptional Regulatory Map (PTRM) (http://plantregmap.gao-lab.org/binding_site_prediction.php) with *p* ≤ 1e-6 (Tian et al., 2020). The transcription factor regulatory network was constructed and visualized with the Cytoscape 3.9 software (Kohl et al., 2011).

### *PeCER* Genes Expression Analyses of Different Tissues

The expression profiles of the 34 identified *PeCER* genes in different tissues of yellow and purple passion fruit cultivars were evaluated through the available public data. The RNA-Seq raw reads were downloaded from the NCBI Sequence Read Archive database (http://www.ncbi.nlm.nih.gov/sra) with the following accession numbers: SRP150688 (Xu et al., 2019) and PRJNA634206 (Wu et al., 2021). The passion fruit sample information for the NCBI raw reads was as follows: peel tissue samples were from yellow (*P. edulis*. Flavicarpa cv Huangjin) and purple (*P. edulis*. Sims cv Tainong) cultivars in the ripening stage, and pulp samples were from fruit developmental stages (fruitlet, green, version, and ripening stages). Root tissue samples were from the purple passion fruit Pingtan-1 cold-tolerant cultivar in two cultivation areas: limestone (L) and sandy dolomite (D) rocky desertification areas. Leaf tissue samples were from yellow Huangjinguo (HJG) cold-sensitive and purple Tainong-1 cold-tolerant cultivars under normal temperature (NT) and chilling stress (CS) conditions.

RNA-Seq raw reads were quality-controlled and filtered with the fastp package (Chen S. et al., 2018) and mapped to the passion fruit reference genome (Ma et al., 2021) using the HISAT2 package (Kim and Langmead, 2015) in Ubuntu wslv 20.04.3 (https://ubuntu.com/wsl). The sequence alignment map (SAM) files were transformed to binary alignment map (BAM) and sorted with the Samtools package (Li et al., 2009). Fragments per kilobase of exon per million mapped fragments (FPKM) values were calculated using the limma and edgeR (Law et al., 2016). Since the FPKM expression values of different tissues of passion fruit were quite different, the FPKM values were converted to log^2^FC (FC = fold change), and heatmaps were constructed using the TBtools software.

### Plant Material

To study the expression profiles of selected *PeCER* genes under drought stress and normal conditions, seeds of two commercial passion fruit cultivars yellow (*P. edulis*. Flavicarpa cv Huangjin) and purple (*P. edulis*. Sims cv Tainong) were grown in plastic pots filled with peat moss and soil (2:1 ratio). Greenhouse temperature was 25 ± 2°C, photoperiod was 16/8 h, light/dark, and relative humidity was 75%. One-month-old passion fruit seedlings were subjected to dehydration for 10 days and rewatered. Leaf, root, and stem samples from each cultivar with three biological replicates were taken and immediately frozen in liquid nitrogen and were stored at −80°C for further use. Samples from normally watered plants were taken as control. For *F. kyushuense* fungal biotic stress samples, fruits of both cultivars were obtained from a commercial orchard located in Fujian province, China (23°48035.200 N and 117°07008.100 E). Fruits were surface-sterilized and inoculated with *F. kyushuense* pathogenic fungus by following the protocol mentioned in our previous publication (Rizwan et al., 2021). Peel samples from the infected areas were collected at 9th and 12th day post-inoculation (dpi), and fruits without infections were used as control.

### RNA Extraction and qRT-PCR Analysis

Total RNA was isolated from frozen samples using a Tiangen mini-RNA extraction kit (Tiangen, China) following the manufacturer's instructions and was quantified with a Thermo Fisher Scientific NanoDrop 2000 UV-Vis spectrophotometer (United States). The first strand of complementary DNA (cDNA) was synthesized using 1 μg of total RNA by Takara PrimeScript™ RT Reagent Kit with a gDNA eraser (TAKARA, China), and cDNA was diluted five times with double distilled water (ddH_2_O). The quantitative real-time polymerase chain reaction (qRT-PCR) was carried out on LightCycler® 96 (Roche Applied Science, Penzberg, Germany) in a 20-μl total reaction mixture, which was composed of 10 μl of a TB Green premixed enzyme solution (TAKARA, China), 1 μl each of the forward and reverse primers (100 μM), 1 μl cDNA, and 7 μl ddH_2_O. Passion fruit genes encoding *Pe60S, PeTIF* (transcription initiation factor), and histone were used as the reference genes for internal control (Munhoz et al., 2015). The qRT-PCR reaction was performed as described below, including preincubation at 95°C for 30 s, followed by 45 cycles at 95°C for 10 s, and 60°C for 30 s. Each reaction was performed with three biological replicates, and relative gene expression levels were normalized with the *Pe60S* gene and calculated using the 2^−Δ*ΔCT*^ method (Schmittgen and Livak, 2008). The details of all the primers used in this study can be found in Supplementary Table S1.

### Subcellular Localization of PeCER32 Protein

To validate the predicted results of the subcellular localization of PeCER proteins, a transient expression assay of the PeCER32 protein was performed in onion epidermal cells with a pCAMBIA1302 vector containing a cauliflower mosaic virus 35S (CaMV35S) promoter and a green fluorescent protein (GFP) tag in the upstream region of the multiple cloning sites (MCS). The full-length CDS (930 bp) of PeCER32 without stop codon was amplified by PCR using the following primers: 5′-ACGGGGGACTCTTGACCATGGATGAAGGTGACTGTAGTTTCTCGC-3′ (*NcoI*) and 5′- TCTCCTTTACTAGTCAGATCTCAGGAATGGGGGCAGTATAATCCA-3′ (*BglII*) (restriction sites are underlined), and cloned into the pMD™ 19-T vector (Cat# 6013; TAKARA, Shiga, Japan). Sequencing (Sangon Biotech Co., Ltd., Shanghai, China) was performed to confirm the positive clones, which were digested with *NcoI* and *BglII* restriction enzymes (TAKARA, China). ClonExpress II One Step Cloning Kit (Cat# C112; Vazyme Biotech Co., Ltd., Nanjing, China) was used for ligation into the *NcoI*-*BglII-*digested pCAMBIA1302 vector and transferred to *Agrobacterium tumefaciens* EHA105 strain for subsequent infection. The resulting plasmids were named CaMV35S-PeCER32-GFP and empty vector CaMV35S-GFP, and they were successfully transformed into onion epidermal cells with the agroinfiltration method. GFP signals were detected and visualized with a laser scanning confocal microscope (Olympus, Tokyo, Japan; FV1200) after 24-72 h of agroinfiltration (Xu et al., 2014).

### Statistical Analysis

All statistical analyses were performed by one-way analysis of variance (ANOVA) and figures were generated with GraphPad Prism 8.0.1 (https://www.graphpad.com/scientific-software/prism). Student's *t*-test (*p* < 0.05) was conducted to compare the samples of yellow and purple passion fruits tissues under controlled and stressed condition.

## Results

### Identification and Physicochemical Properties of *CER* Genes in Passion Fruit

In this study, the known CER protein sequences from different species were blasted with passion fruit genome proteins and after removing redundant, repetitive, and unrecognized sequences, finally, 34 *CER* genes were identified in the passion fruit genome with computational tools. The passion fruit *CER* genes were named from *PeCER*1 to *PeCER*34 based on their positions on chromosomes (Table 1). The *PeCER* genes were unevenly distributed on eight out of nine passion fruit chromosomes. Chromosome number 1 contained a comparatively high number of *PeCER* genes (12 genes) followed by chromosome 6 (6 genes), chromosome 5 (5 genes), chromosomes 2 and 8 (4 genes on each); chromosomes 3, 5, and 9 contained only one *PeCER* gene (Table 1, Supplementary Figure S1). The detailed information of all the 34 *PeCER* genes is available in Table 1, and protein sequences have been provided in Supplementary Table S2. The protein analysis shows that the length of the PeCER protein varied from 182 (PeCER15) to 1,718 bp (PeCER13), that the CDS length ranged from 549 (*PeCER*15) to 5,157 bp (*PeCER*13), and that the protein molecular weight ranged from 20.64 (PeCER15) to 199.26 KDa (PeCER13) (Table 1). The isoelectric point of proteins ranged from 5.92 (PeCER23) to 10.07 (PeCER2), the protein instability index varied from 30.11 (PeCER3) to 52.21 (PeCER30), and the aliphatic index of proteins varied from 82.11 (PeCER30) to 110.12 (PeCER28). The GRAVY ranged from −0.25 (PeCER16) to 0.383 (PeCER21). The number of exons varied from 1 (PeCER6, PeCER8, and PeCER9) to 28 (PeCER13). The subcellular location prediction showed that most of the PeCER proteins were associated with the plasma membrane except for PeCER2, PeCER3 and PeCER15 (mitochondrial), and PeCER22 and PeCER23 (cytoplasmic) (Table 1).

**Table 1:** Physicochemical features of *PeCER* genes.

**Gene ID**	**Gene name**	Chr*	**Genomic position**	**CDS (bp)**	A.A*(bp)	M.W*	pl*	ll*	Ai*	GR-AVY*	E:I*	SCL*
*PeCER*1	ZX.01G0006120	1	16013784:16016240-	741	246	28.2	7.4	46.1	96.8	0.02	5:4	pm
*PeCER*2	ZX.01G0019790	1	26796949:26798567+	846	281	32.6	10.1	32.1	84.0	−0.41	6:5	mit
*PeCER*3	ZX.01G0019860	1	26843466:26844659+	849	282	32.0	9.8	30.1	104.4	−0.10	5:4	mit
*PeCER*4	ZX.01G0024040	1	30497870:30500240+	1,533	510	57.4	9.4	40.9	95.4	−0.08	2:01	pm
*PeCER*5	ZX.01G0024770	1	31123019:31125903+	774	257	29.7	7.3	37.3	101.6	0.15	2:1	pm
*PeCER*6	ZX.01G0031680	1	36909208:36910698+	1,491	496	56.1	9.4	40.5	100.2	0.02	1:00	pm
*PeCER*7	ZX.01G0039150	1	41610479:41619444–	4,395	1,464	163.5	6.1	43.1	104.6	0.21	21:19	pm
*PeCER*8	ZX.01G0039870	1	42004537:42005907+	1,371	456	51.2	8.8	34.1	91.7	−0.06	1:00	pm
*PeCER*9	ZX.01G0112910	1	220335704:220337194+	1,491	496	56.1	9.0	40.1	97.7	0.01	1:00	pm
*PeCER*10	ZX.01G0116460	1	222578824:222581518+	1,572	523	59.3	9.1	38.9	89.3	−0.14	2:01	pm
*PeCER*11	ZX.01G0124790	1	231570306:231574170–	879	292	33.8	6.6	41.5	90.8	0.03	5:4	pm
*PeCER*12	ZX.01G0136790	1	267272100:267274802–	933	310	36.0	9.6	51.4	86.4	−0.11	4:03	pm
*PeCER*13	ZX.02G0014730	2	59578861:59589952+	5,157	1,718	199.3	8.8	39.0	93.3	−0.07	28–29	pm
*PeCER*14	ZX.02G0015130	2	59791229:59797692–	2,367	788	90.9	7.0	40.4	85.1	−0.21	14:13	pm
*PeCER*15	ZX.02G0015160	2	59820344:59821338–	549	182	20.6	9.5	38.9	107.6	−0.17	5:4	mit
*PeCER*16	ZX.02G0018030	2	61499311:61505027+	1,698	565	63.6	8.6	40.5	78.2	−0.25	9:8	pm
*PeCER*17	ZX.03G0021830	3	111603305:111606334+	711	236	27.4	8.8	37.6	86.7	0.14	6:5	pm
*PeCER*18	ZX.04G0004390	4	5557716:5565015+	1,968	655	73.5	8.9	40.9	95.9	−0.06	5:04	pm
*PeCER*19	ZX.04G0008120	4	18732041:18739420+	1,584	527	59.4	6.1	47.5	104.3	0.01	11:10	pm
*PeCER*20	ZX.04G0030740	4	111909920:111921418–	4,173	1,390	155.2	8.9	44.6	93.3	0.08	19:18	pm
*PeCER*21	ZX.04G0031300	4	113389251:113392492+	1,263	420	48.4	8.8	43.5	101.0	0.38	8:7	pm
*PeCER*22	ZX.04G0031310	4	113392969:113394946+	675	224	25.0	8.5	33.4	93.1	−0.06	5:4	cyt
*PeCER*23	ZX.05G0019760	5	112330539:112336467+	2,028	675	76.2	5.9	35.7	86.5	−0.21	19:18	cyt
*PeCER*24	ZX.06G0000900	6	3463805:3474069–	2,904	967	108.4	9.9	51.1	84.7	−0.21	16:15	pm
*PeCER*25	ZX.06G0022930	6	107622892:107626542–	1,905	634	73.8	8.5	36.1	95.0	0.01	10:9	pm
*PeCER*26	ZX.06G0022950	6	107650045:107652816–	1,644	547	63.5	8.8	34.3	94.4	−0.03	7:6	pm
*PeCER*27	ZX.06G0023310	6	107833067:107834602–	1,536	511	57.0	9.2	38.3	96.2	−0.01	1:00	pm
*PeCER*28	ZX.06G0024510	6	108511963:108517390–	3,390	1,129	126.0	5.9	36.8	110.1	0.34	8:07	pm
*PeCER*29	ZX.06G0027150	6	110954426:110966330–	4,227	1,408	157.6	9.1	45.9	91.1	0.07	15:14	pm
*PeCER*30	ZX.08G0019030	8	49562396:49563948+	738	245	27.9	9.7	52.2	82.1	−0.04	5:4	pm
*PeCER*31	ZX.08G0019680	8	50041436:50043895–	1,080	359	42.4	8.8	48.5	95.9	0.00	4:3	pm
*PeCER*32	ZX.08G0029780	8	57784308:57787580–	933	310	36.2	9.6	48.7	85.5	−0.08	4:03	pm
*PeCER*33	ZX.08G0032230	8	61391202:61394104–	738	245	28.2	7.2	37.6	92.3	0.05	3:2	pm
*PeCER*34	ZX.09G0020720	9	69749686:69754295–	711	236	27.5	9.0	37.6	82.5	0.10	6:5	pm

Chr^*^, chromosome NO; A.A^*^, amino acid/protein length; M.W^*^, molecular weight (KDa); pI^*^, isoelectric point; ll^*^, instability index; Ai^*^, aliphatic index; GRAVY^*^, grand average of hydropathicity; E:I^*^, number of exons:introns; SCL^*^, subcellular localization; pm^*^, plasma membrane; mit, mitochondrial; cyt, cytoplasmic. Positive (+) and negative (–) signs indicate the presence of a gene on the positive and negative strands of that particular marker at genome location.

### Phylogenetic Analysis of *CER* Genes

**Figure 1:**
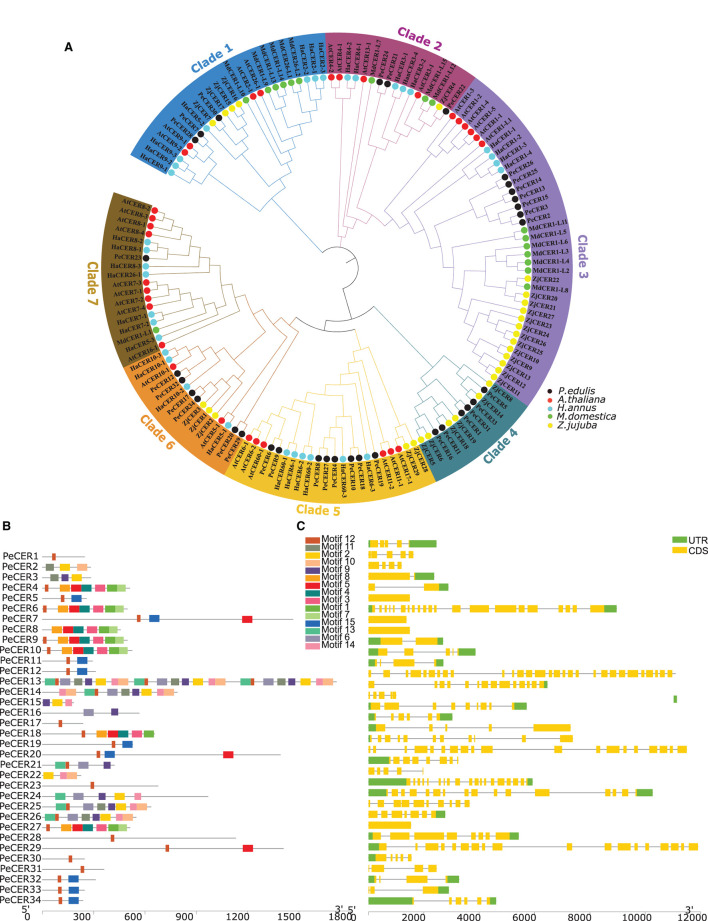
**(A)** Unrooted neighbor-joining phylogenetic tree among *P. edulis, A. thaliana, M. domestica, H. annus*, and *Z. jujuba* CER amino acid sequences with 1,000 bootstraps constructed with MEGA software v10.1.8. The protein sequences of PeCER, AtCER, HaCER, MdCER, and ZjCER are represented by black, red, aqua, green, and yellow color circles, respectively. **(B)** Conserved motifs were represented *via* boxes, and different colors represent different motifs. **(C)** *PeCER* gene structures; yellow color indicates the exons, green color shows the untranslated 5′ and 3′-regions, and gray color indicates the introns.

One hundred and forty-four CER protein sequences from *P. edulis* (PeCER), *A. thaliana* (AtCER), *M. domestica (*MdCER), *H. annus* (HaCER), and *Z. jujuba* (ZjCER) were used to assess the evolutionary relationship and construct the phylogenetic tree (all the protein sequences used for the phylogenetic tree has been provided in Supplementary Table S2). The protein sequences were aligned, and a neighbor-joining tree was constructed by grouping into seven clades based on sunflower *HaCER* (Ahmad et al., 2021) (Figure 1A). The results showed that clade 1 comprised of 24 *CER* genes (3 *PeCER*s, 4 *AtCERs*, 7 *HaCERs*, 6 *MdCERs*, and 4 *ZjCERs*); clade 2 comprised of 16 genes (3 *PeCER*s, 4 *AtCERs*, 1 *ZjCERs*, 3 *MdCERs*, and 5 *HaCERs*); clade 3 comprised of 37 genes (7 *PeCER*s, 6 *AtCERs*, 4 *HaCERs*, 7 *MdCERs*, and 13 *ZjCERs*); clade 4 comprised of 12 genes (6 *PeCER*s and 6 *ZjCERs*); clade 5 comprised of 22 genes (8 *PeCER*s, 6 *AtCERs*, 6 *HaCERs*, and 2 *ZjCERs*); clade 6 comprised of 15 genes (6 *PeCER*s, 4 *HaCERs*, 2 *AtCERs*, and 3 *ZjCERs*); clade 7 comprised of 18 genes (1 *PeCER*, 9 *AtCERs*, 7 *HaCERs*, and 1 *MdCER*) (Figure 1A). Each clade varied in the number of genes; among them, clade 2 was found to be the largest group containing the highest number of *CER* genes (37 genes) followed by clade 1 (24 genes), whereas clade 4 was the smallest group that contained only 12 *CER* genes (Figure 1A). *PeCER* genes also varied in their unequal distribution among the different clades such as the highest number of *PeCER* genes (8 genes) found in clade 5 followed by clade 3 (7 genes), and clade 7 contained only 1 *PeCER* gene (Figure 1A). These alignment and evolutionary results revealed that the PeCER proteins shared greater homology with *A. thaliana* and *Z. jujuba* compared with *H. annus* and *M. domestica* CER proteins (Figure 1A). In terms of pairwise gene similarity percentage index in the *PeCER* genes, the similarity index also varied from 2.66 (*PeCER*7/*PeCER*22) to 91.54% (*PeCER*6/*PeCER*9). The results showed that the PeCER6 and PeCER9 proteins showed the highest similarity of 91.54% and were consistent with the phylogenetic results as grouped in the same clade 5 (Figure 1A, Supplementary Table S3).

### *PeCER* Gene Structure and Motif Analyses

The conserved motifs and exon-intron organization of gene structure were analyzed to further understand the development of the *PeCER* gene family. Furthermore, the conserved motifs among 34 *PeCER* genes were predicted using the online MEME suit, and the range of conserved motifs varied from 1 to 24 (Figure 1B). Overall, a total of 15 conserved motifs were predicted among the 34 PeCER proteins (Supplementary Table S4). The majority of *PeCER* genes (12 genes) have 8 motifs followed by 3 motifs (4 genes). *PeCER*7 contains 11 motifs, and *PeCER* 15 and *PeCER*16 contain 9 motifs. A maximum of 24 and 10 motifs were recognized in *PeCER*13 and *PeCER*14 respectively, whereas *PeCER*1, *PeCER*17, *PeCER*23, *PeCER*28, *PeCER*30, and *PeCER*31 contained only 1 motif (Figure 1B, Supplementary Table S4). The genomic structural analysis for exon-intron position in the 34 *PeCER* genes revealed that the number of exon-intron in the identified *PeCER* genes varied from 1 to 29 (Figure 1C). Most of the *PeCER* genes contained more than 5 exons, whereas genes such as *PeCER*13, *PeCER*7, *PeCER*20, and *PeCER*24 contained 29, 21, 19, and 16 exons, respectively. For instance, the *PeCER*6, *PeCER*8, and *PeCER*9 genes contained only 1 exon and no intron (Figure 1C). These findings stated that variation of motifs and exon-intron during the evolutionary process occurred instantaneously in *PeCER* gene family development, and that *PeCER* genes that contain the same features may have the same function.

### *PeCER* Gene *cis*-Regulatory Element Analyses

**Figure 2:**
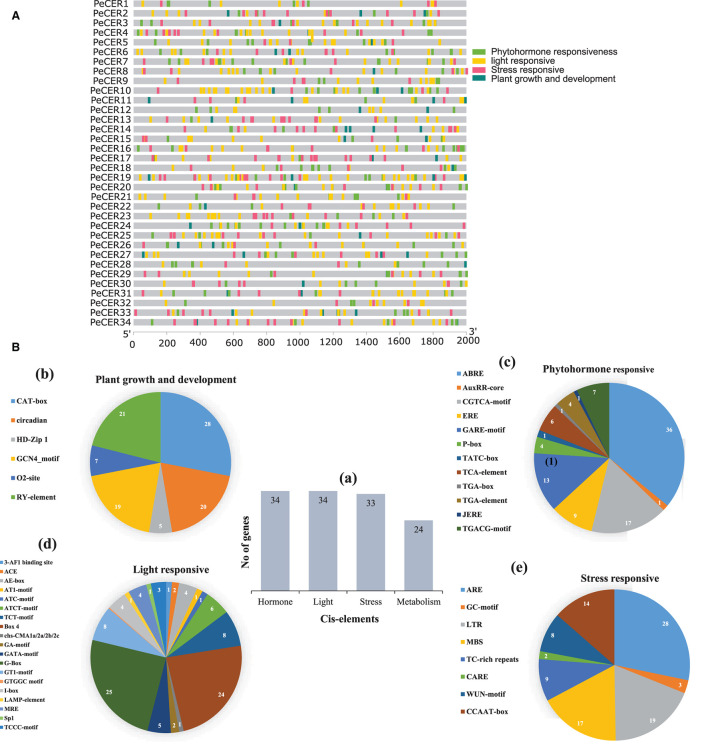
**(A)** Cis-regulatory element analysis on *PeCER* genes. **(B)** (a) Sum number of cis-elements and *PeCER* genes involved in four categories. Percentage (%) ratio of numerous *cis*-elements from each category is presented in pie charts; (b) plant growth and development-responsive, (c) phytohormone-responsive, (d) light-responsive, (e) stress-responsive. Different colors indicate different cis-acting elements and their ratio present in *PeCER* genes.

To further understand the possible role of *PeCER* genes in response to biotic and abiotic stresses, the *cis*-regulatory elements of *PeCER* genes from 2,000 bp upstream promoter regions were analyzed (Figure 2). The promoters of *PeCER* genes mainly comprised four categories of *cis*-regulatory elements including plant growth- and development-responsive (six different types of *cis*-elements including CAT-box, circadian, GCN4-motif, O2-site, RY-element, and CCGTCC motif), phytohormone-responsive (twelve different types of *cis*-elements including ABRE, AuxRR-core, CGTCA-motif, ERE, GARE-motif, P-box, TATC-box, TCA-element, TGA-box, TGA-element, JERE, and TGACG-motif), light-responsive (nineteen different types of *cis*-elements including 3-AF1 binding site, ACE, AE-box, AT1-motif, ATC-motif, ATCT-motif, TCT-motif, Box 4, chs-CMA1a/2a/2b/2c, GA-motif, GATA-motif, G-Box, GT1-motif, GTGGC motif, I-box, LAMP-element, MRE, Sp1, and TCCC-motif), and stress-responsive including anaerobic induction, MYB binding site, and wound and temperature-responsive, (eight different types of *cis*-elements: ARE, GC-motif, LTR, MBS, TC-rich repeats, CARE, WUN-motif, and CCAAT-box) elements (Figure 2A, Supplementary Table S5). In terms of *cis*-element categories, the light-responsive category contains the maximum number of *cis*-elements followed by phytohormone-responsive and stress-responsive, while the lowest number of *cis*-elements was found in the plant growth and development-responsive category (Figure 2B). In addition, the *PeCER* genes were classified based on the number of genes involved in each category (Figure 2Ba). It was found that all the 34 *PeCER* genes were involved in phytohormones and light-responsive, 33 *PeCER* genes were involved in stress-responsive, while only 24 *PeCER* genes were involved in plant growth and development responsiveness (Figure 2Ba). These results proposed that transcript profiling of *PeCER* may vary among hormones and stress-responsive. The plant growth and development category was further classified into four subcategories (meristem, metabolism, seed related, and circadian). Meristem-related *cis*-elements include CAT-box and GCN4-motifs, which belong to meristem expression.

The metabolism-related *cis*-element includes O2-sit and seed, and the circadian-related *cis*-element includes RY-element. In the plant growth and development category, the highest number of cis-elements (28%) consisted of CAT-box, followed by circadian (20%), and the lowest (5%) consisted of HDzip1 (Figure 2Bb). The phytohormone-responsive category comprised of ABRE (abs*cis*ic acid-responsive), AuxRR-core (auxin-responsive), ERE (ethylene-responsive), GARE-motif, P-box, TATC-box (gibberellin responsive); TCA-element (salicylic acid-responsive); TGA-element and TGACG-motif (methyl jasmonate responsive) *cis*-elements. The highest number of *cis*-elements from the phytohormone category comprised of ABRE at 36% followed by CGTCA-motif at 17%, whereas the AuxRR-core, TATC box, and TGA box were the lowest, containing only 1% *cis*-elements (Figure 2Bc). The light-responsive category contained the 3-AF1 binding site, ACE, AE-box, AT1-motif, ATC-motif, ATCT-motif, TCT-motif, Box 4, chs-CMA1a/2a/2b/2c, GA-motif, GATA-motif, G-Box, GT1-motif, I-box, LAMP-element, MRE, Sp1, and TCCC-motif *cis*-elements. The highest light-responsive category comprised of only G-Box (25%) followed by Box-4 (24%), while AT1-motif and ATC-motif were the lowest and comprised of only 1% (Figure 2Bd). The stress-responsive category contained ARE, GC-motif, LTR, MBS, TC-rich repeats, CARE, WUN-motif, and CCAAT-box *cis*-elements; among which, ARE comprised the highest number (28%) followed by LTR and MBS (19 and 17%), while CARE contained only 2% (Figure 2Be). The detailed information about *cis*-regulatory elements in the passion fruit *CER* genes has been provided in Supplementary Table S5.

### Synteny Analysis of *PeCER* Genes

**Figure 3:**
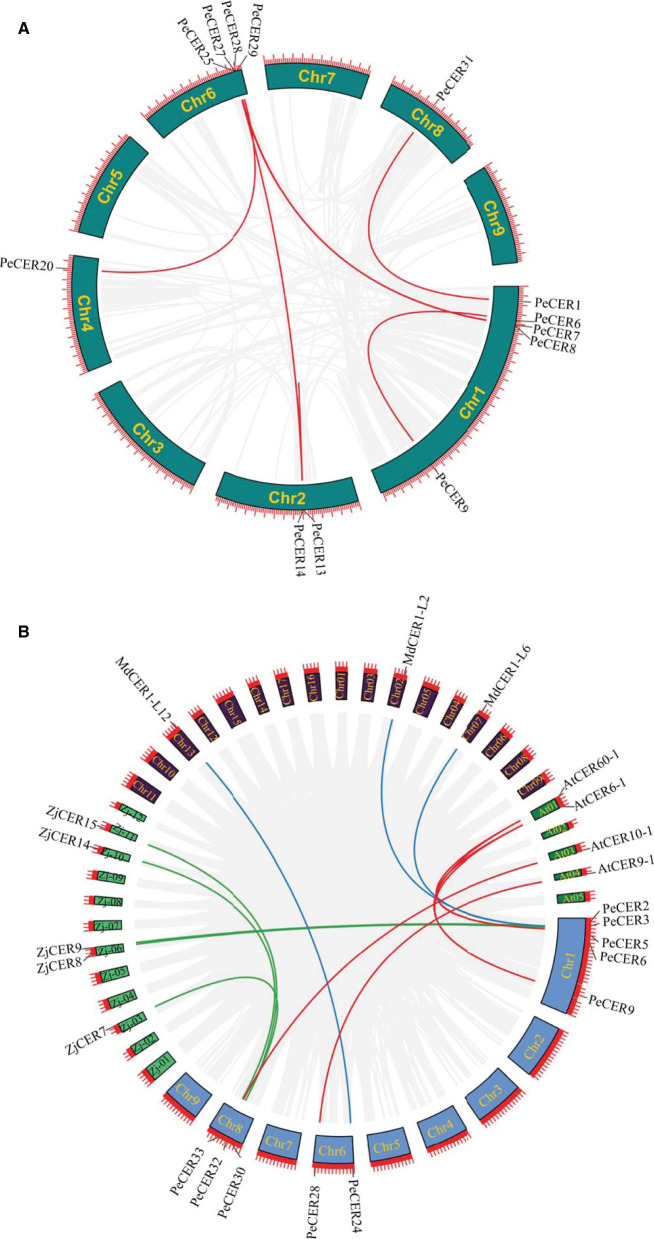
Circos illustrations of *PeCER* gene duplication. **(A)** Gene duplication of *CER* genes in *P. edulis*. The background gray lines show all the syntenic blocks in the passion fruit genome, and the red lines show segmental or tandem duplication link regions among the *PeCER* genes. The approximate location of *PeCER* genes were labeled with a short gray line outside with gene names. **(B)** Orthologous of *P. edulis CER* genes with *A. thaliana, M. domestica*, and *Z. jujuba CER* genes. Chromosomes of *P. edulis are* represented with Chr1-Chr9, *A. thaliana* with At01-At05, *M. domestica* with Chr01-Chr17, and *Z. jujuba* with Zj-01 to Zj-12. The background gray lines show all the syntenic blocks in different genomes, blue lines represent the syntenic relationship among *P. edulis* and *M. domestica CER* genes, red lines represent the syntenic relationship among *P. edulis* and *A. thaliana* genes, and green lines represent the syntenic relationship among *P. edulis* and *Z. jujuba* genes.

The expansion and evolutionary mechanism of the *PeCER* gene family in the passion fruit genome and genomes of other species were further investigated by synteny analysis. The tandem and segmental duplication analysis of the *PeCER* genes was performed to elucidate the duplication of the *PeCER* genes. There were eight duplicated *PeCER* gene pairs, among which six gene pairs were segmentally duplicated on chromosomes 1, 2, 4, 6, and 8, whereas two *PeCER* gene pairs were tandem-duplicated on chromosomes 1 and 2. *PeCER*13 and *PeCER*25 were duplicated twice (Figure 3A, Supplementary Table S6). These findings suggested that gene duplication has played an important role in *PeCER* gene family development in the passion fruit genome.

To determine the selection pressure and evolution rate among duplicated genes, Ka/Ks ratio is an indicator. Generally, if Ka/Ks ratio is more than 1, it indicates that genes have undergone a positive selection, a ratio <1 indicates purifying selection, while a ratio equal to 1 indicates neutral selection. The details about Ka and Ks values and Ka/Ks ratios of duplicated *PeCER* gene pairs are shown in Supplementary Table S6. Overall, all the segmental and tandem duplicated *PeCER* gene pairs exhibited a Ka/Ks ratio <1, indicating that all the duplicated *PeCER* genes underwent purifying selection (Supplementary Table S6). Furthermore, the time of divergence among the duplicated genes was measured based on a substitution rate of 6.38 × 10^−9^ substitutions per site per year (Ma et al., 2021). The results showed that the rate of divergence process among the tandem and segmental *PeCER* genes was estimated to be 1.12 to 20.78 mya (Supplementary Table S6). It can be concluded that the evolutionary mechanism of *PeCER* genes shows maintenance in the process of passion fruit domestication.

In addition, a comprehensive synteny analysis of *P. edulis CER* genes with *CER* genes of other species, including *A. thaliana, M. domestica*, and *Z. jujuba*, was performed by alignment and chromosomal localization (Supplementary Table S7). The results showed that out of the 34 *PeCER* genes, 10 *PeCER* were paired with 12 *CER* genes from the above-mentioned species, and that the 10 *PeCER* genes were located on passion fruit chromosomes 1, 6, and 8 (Figure 3B, Supplementary Table S8). The presence of six *PeCER* gene pairs with *AtCER* (*PeCER*9:*AtCER60*-1, *PeCER*9:*AtCER6*-1, *PeCER*6:*AtCER6*-1, *PeCER*6:*AtCER60*-1, and *PeCER*32:*AtCER10*-1) (Figure 3B, Supplementary Table S8), three *PeCER* gene pairs with *MdCER* (*PeCER*3:*MdCER1*-L2, *PeCER*2:*MdCER1*-L6, and *PeCER*24:*MdCER1*-L12), and five *PeCER* gene pairs with *ZdCER* (*PeCER*3:ZjCER9, *PeCER*5:ZjCER8, *PeCER*30:ZjCER7, *PeCER*33:ZjCER14, and *PeCER*30:ZjCER15) was detected and visualized with Circos (Figure 3B, Supplementary Table S8). Overall, the *PeCER* and *AtCER* genes showed a greater degree of synteny than the other species, indicating that they might belong to the same ancestors with the same functions, which required further study.

**Figure 4:**
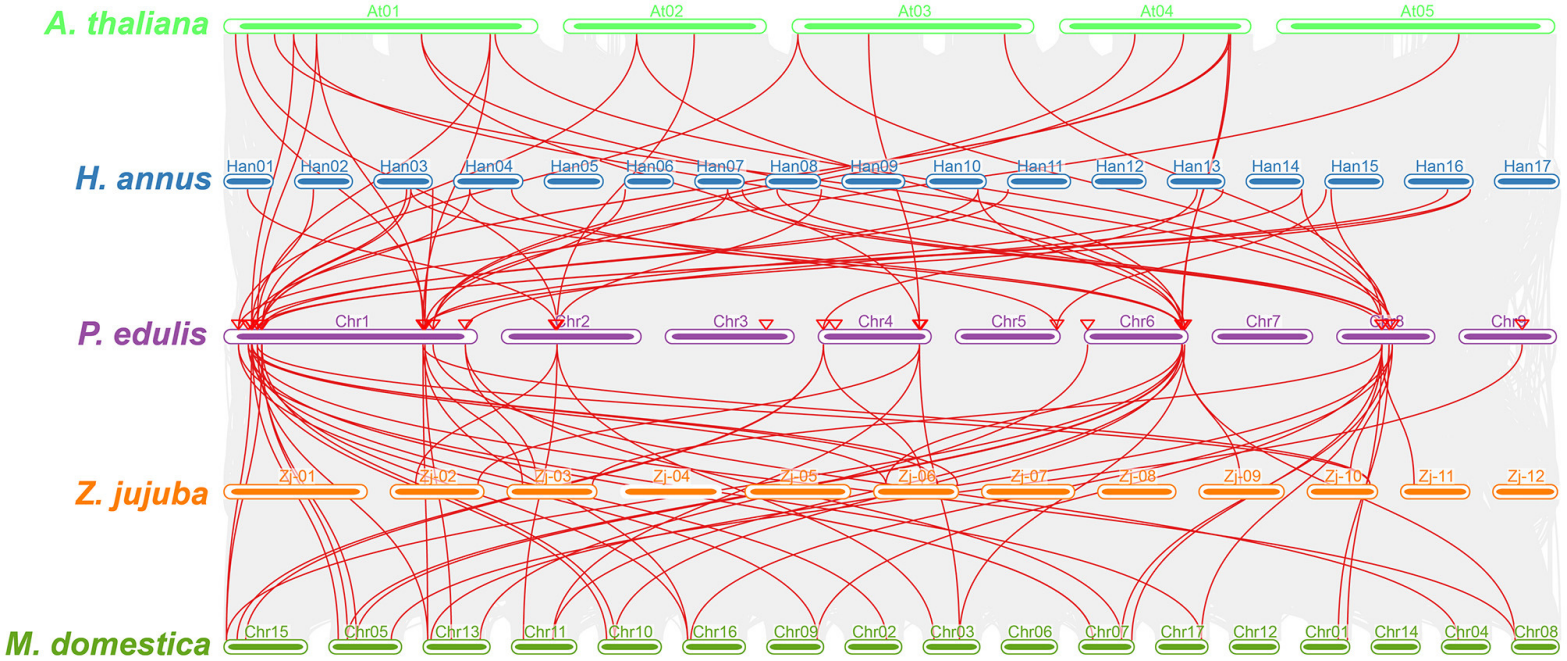
Multicollinearity analysis of the P. edulis CER genes with genomes of the *A. thaliana, M. domestica, H. annus*, and *Z. jujuba* species. The red lines represent the CER syntenic genes among *P. edulis, A. thaliana, M. domestica, H. annus*, and *Z. jujube* genomes, and the gray lines in the background represent all orthologous genes from the genomes of *P. edulis* and other species.

Furthermore, a multicollinearity analysis of *P. edulis CER* genes with genomes of *A. thaliana, M. domestica, H. annus*, and *Z. jujuba* species was performed to reveal the robust orthologs in the genomes of these species. Multiple collinear gene pairs between the aforementioned species were found to be inferred and have undergone lineage-specific amplification of genetic copies (Figure 4). The multicollinearity results showed that the highest collinearity was found between *P. edulis* with *M. domestica* (43 collinear genes) followed by *P. edulis* with *H. annus* and *A. thaliana* (26 collinear genes), whereas the least collinearity was found between *P. edulis* with *Z. jujuba* (16 collinear genes) (Figure 4, Supplementary Table S9). *P. edulis* chromosome 1 shared the maximum collinear genes among *M. domestica* (20 genes), *H. annus* (14 genes), *A. thaliana* (13 genes), and *Z. jujuba* (7 genes) (Figure 4, Supplementary Table S9). These findings suggest that the *CER* genes were conserved and might have the same ancestors besides the duplication or loss of *CER* genes.

### Protein-Protein Interaction and 3D Modeling of PeCER

**Figure 5:**
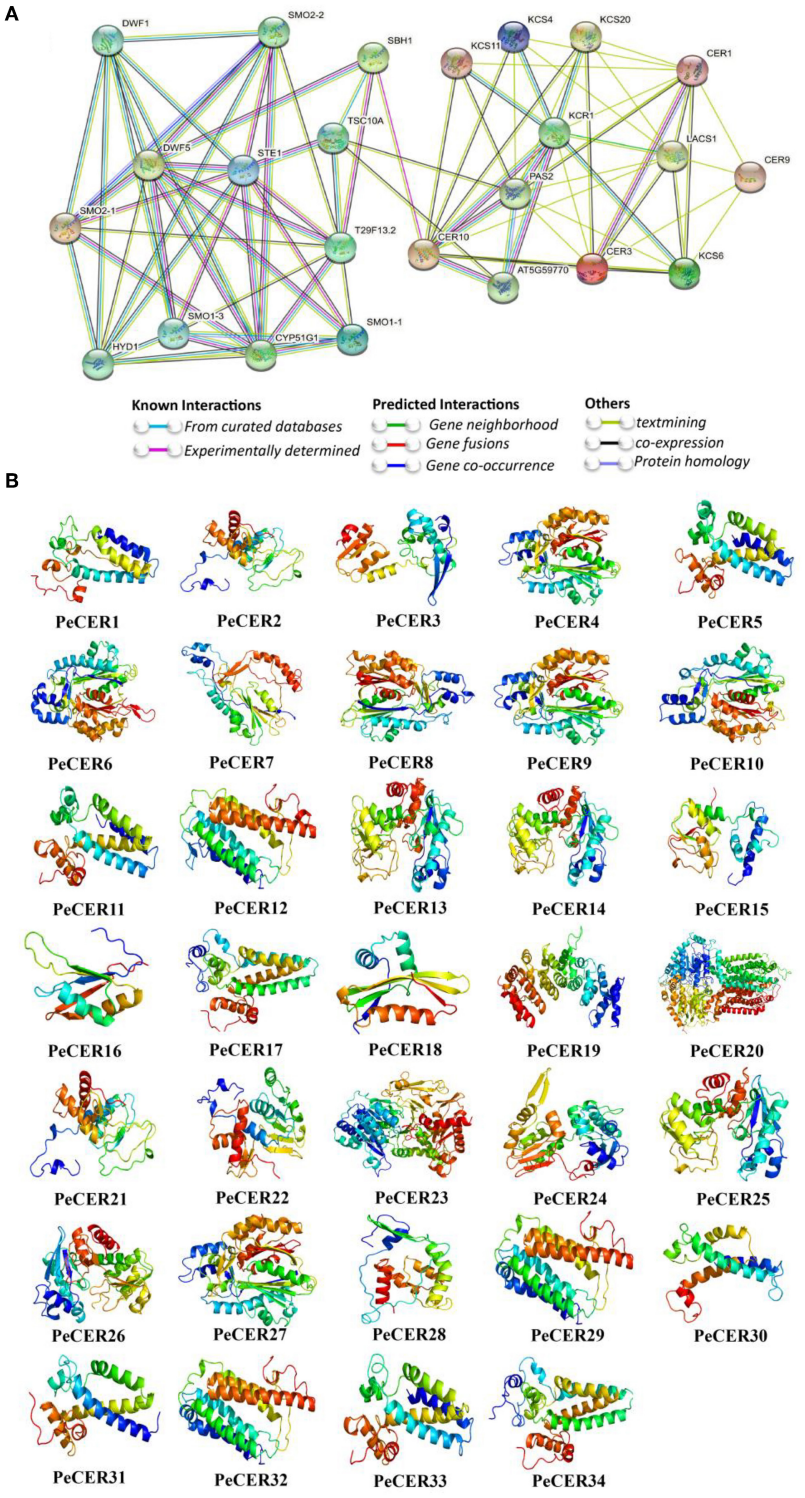
Protein-protein interaction of PeCER proteins based on known *Arabidopsis* protein orthologous. **(A)** The network was constructed using the online STRING software. The proteins were displayed at network nodes with 3D structure of the proteins in nodes, and the line colors indicate different data sources. **(B)** Predicted 3D models of PeCER proteins. 3D models were constructed using the online Phyre2 server with default mode.

The PeCER protein-protein interaction network was constructed based on *Arabidopsis* orthologous proteins. PeCER proteins with highest homologous similarity to *Arabidopsis* proteins were selected as STRING proteins. All the 34 PeCER proteins have an association with known *Arabidopsis* proteins (Figure 5A, Supplementary Table S10). PeCER proteins belonging to different groups may have diverse functions. PeCER2, PeCER3, PeCER13, PeCER14, PeCER15, PeCER25, and PeCER26 were homologous with AtCER1; PeCER21 and PeCER28 with AtCER9; PeCER32 with AtCER10; PeCER 23 with AtLACS1; PeCER22 and PeCER24 with AtCER3, and they all had a strong interaction among them. PeCER1 and PeCER31 were homologous with AtSTE1 and interacted with AtDWF1, AtDWF5, AtSMO2-1, AtSMO2-2, AtSMO2-3, and AtFAH1/T29F13.2 (Figure 5A). PeCER2, PeCER3, PeCER14, PeCER15, PeCER25, and PeCER26 were homologous with AtCER1; PeCER4 and PeCER8 were homologous with AtKCS4; PeCER6 and PeCER9 with AtKCS6; PeCER18 with AtKCS11; PeCER10 with AtKCS20, and they had a strong interaction between them (Figure 5A). PeCER5 and PeCER33 showed homology with AtSBH1 and interacted with AtCER10, AtDWF5 and AtFAH1/T29F13.2. PeCER proteins and that showed a strong interaction with known *Arabidopsis* proteins and might have similar functions as in *Arabidopsis*. The higher the interaction coefficient, the thicker the line between proteins and vice versa (Figure 5A, Supplementary Table S10).

A protein secondary structure analysis was performed, which generally consists of alpha helices, extended strands, beta turns, and random coils (Supplementary Table S11). Among all the 34 PeCER proteins, alpha helix accounted for the largest percentage of secondary structures ranging from 31.96 to 57.2% (PeCER13, PeCER19) followed by random coils from 25.19 to 40.95% (PeCER19, PeCER24), and extended strands from 11.43 to 25.68% (PeCER33 and PeCER15), whereas beta turns ranged from 1.95 to 9.29% (PeCER28 and PeCER15) (Supplementary Table S11). Additionally, the 3D structures and models of all the 34 PeCER proteins were predicted by the Phyre2 online server with default mode (Figure 5B).

Templates were used for the predicted PeCER protein models such as template c4zr0A was used in the PeCER1, PeCER5, PeCER11, PeCER17, PeCER30, PeCER31 and PeCER33 protein models; template c6jzuA was used in the PeCER2, PeCER3, PeCER13, PeCER14, PeCER15, PeCER21, PeCER22, PeCER24, PeCER25, and PeCER26 protein models; template c4jatB was used in the PeCER6 and PeCER9 protein models; template c4zr0A in the PeCER34 model; template c5mstA in the PeCER23 model; template c7c83A in the PeCER12 and PeCER29 models; template d2cq0a1 in the PeCER16 model; template c1u0mA in the PeCER10 model; templatec6hb2B in the PeCER19 model; template c2d8sA in the PeCER7 and PeCER28 models; template c3wy0A in the PeCER8 model; template c4jatB in the PeCER4 and PeCER27 models; template c7p06A in the PeCER20 model; template c7c83A in the PeCER32 model; template c6z00B in the PeCER18 model. All the PeCER proteins had flexible structures due to the existence of coils (Figure 5B). Based on our results, it was proposed that *CER* genes from individual genomes might be ancestrally similar to each other or initial differences may have been stabilized during long-term domestication, leading to changes in protein structure and function.

### Genome-Wide Prediction of Potential miRNAs Targeting *PeCER* Genes

**Figure 6:**
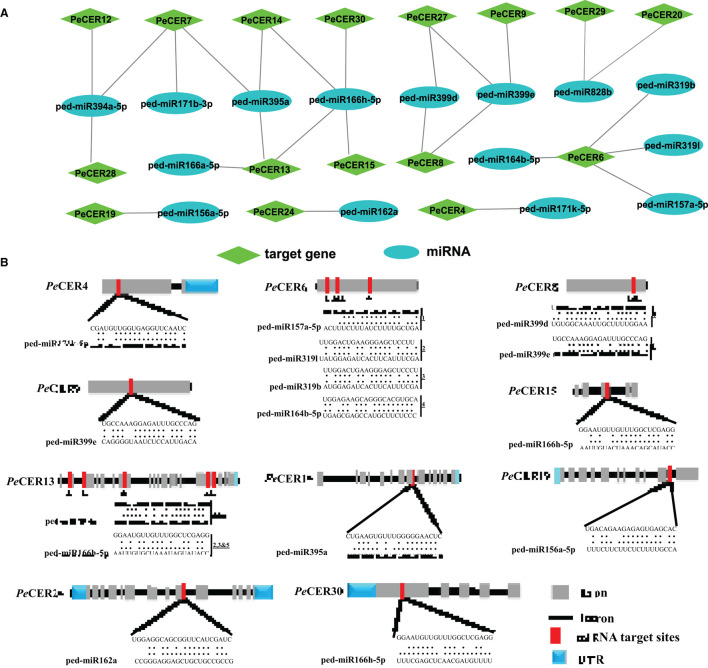
Predicted miRNA targeting *PeCER*s genes. **(A)** Network illustration of predicted miRNA targeting *PeCER* genes. Green hexagon colors represent *PeCER* genes, and bluish ellipse shapes represent miRNAs. **(B)** The schematic diagram indicates the *PeCER* genes targeted by miRNAs.

During the past few decades, various investigations have reported that the miRNAs induced the regulation of stresses, plant development, and signal transduction. Therefore, to better understand the regulatory mechanism of miRNAs involved in the regulation of *PeCER* genes, 12 putative miRNAs targeting 16 *PeCER*s genes were identified, as shown in the network illustration (Figure 6A) and the schematic diagrams indicating the *PeCER* genes targeted by miRNAs (Figure 6B). The detailed information on the putative miRNA targeting sites and the *PeCER* genes has been provided in Supplementary Table S12. The results showed that two members of the ped-miR171 family targeted two genes including *PeCER4* and *PeCER7*; one member of the ped-miR157 family targeted one gene, *PeCER*6; one member of the ped-miR164 family targeted one gene, *PeCER*6; two members of the ped-miR319 family targeted one gene, *PeCER*6; one member of the ped-miR395 family targeted three gene including, *PeCER*7, *PeCER*13, and *PeCER*14; one member of the ped-miR394 family targeted three gene including, *PeCER*7, *PeCER*12, and *PeCER*28; two members of the ped-miR399 family targeted three gene including, *PeCER*8, *PeCER*9, and *PeCER*27; two members of the ped-miR166 family targeted three gene including, *PeCER*13, *PeCER*14, *PeCER*15, and *PeCER*30; one member of the ped-miR828 family targeted two gene including, *PeCER*20 and *PeCER*29; one member of the ped-miR162 family targeted only *PeCER24* gene (Figures 6A,B, Supplementary Table S12).

Overall, the prediction results show that the *PeCER*7 gene was targeted by three miRNA families including, ped-miR395a, ped-miR394a-5p, and ped-miR171b-3p; similarly, the *PeCER*13 gene was also targeted by three miRNA families including, ped-miR166h-5p, ped-miR395a, and ped-miR166a-5, whereas the *PeCER*6 gene was targeted by four miRNA families including, ped-miR157a-5p, ped-miR164b-5p, ped-miR319b, and ped-miR319l (Figures 6A,B, Supplementary Table S12). The expression levels of these predicted miRNAs and their target *PeCER* genes require further studies to better understand their functions in passion fruit.

### Transcription Factor Regulatory Network of *PeCER* Genes

**Figure 7:**
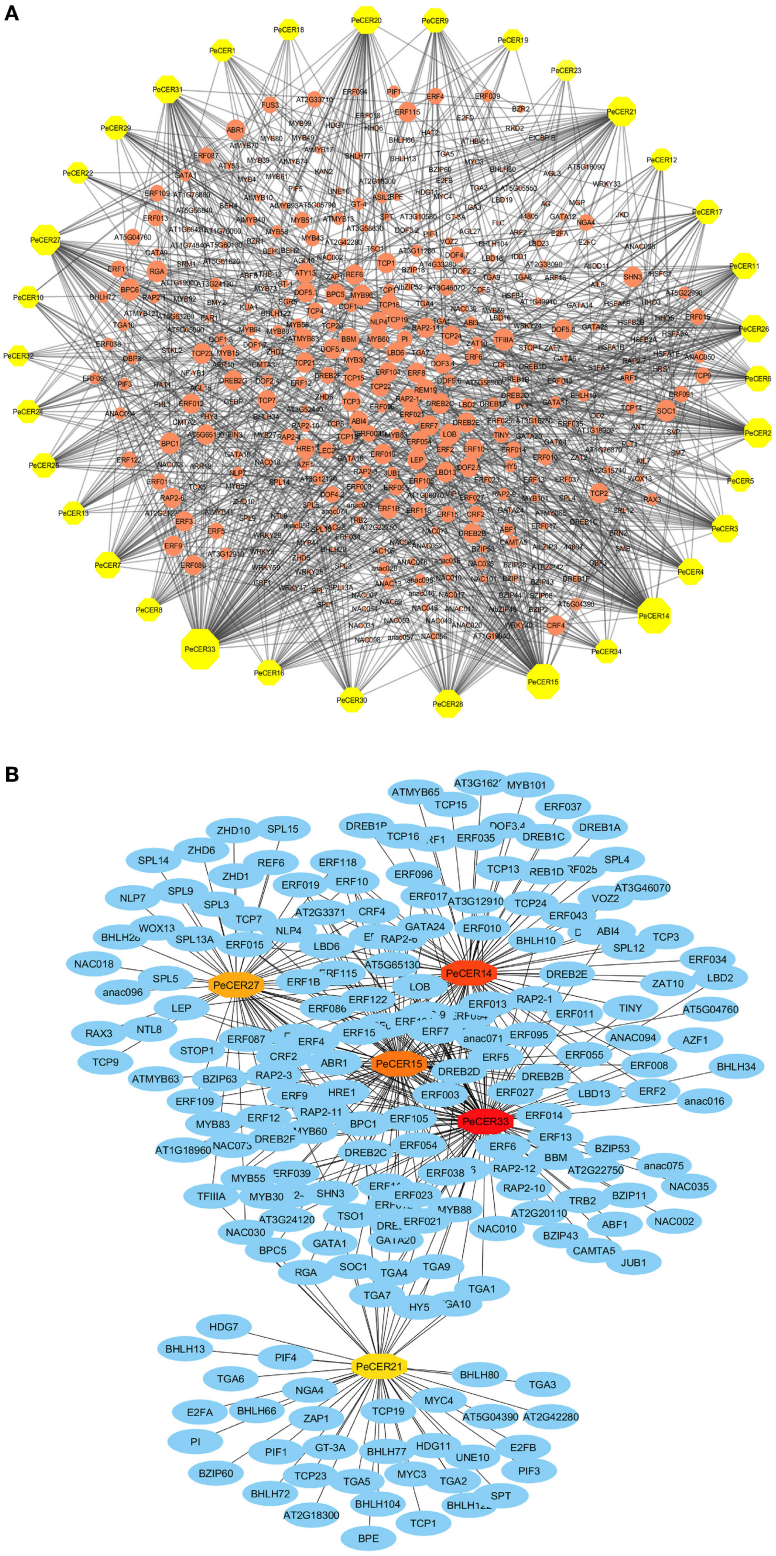
Putative transcription factor regulatory network analysis of the *PeCER* genes. **(A)** Orange peel circular nodes represent transcription factors; yellow octagonal nodes represent *PeCER* genes, and node size represents the degree of interaction between nodes based on degree value. **(B)** Top 5 highly enriched and targeted *PeCER* genes with TFs are shown, blue nodes represent the TFs, dark red to light red colors show the *PeCER* genes, and darker *PeCER* node color shows highly enriched and vice versa.

The potential TF regulatory network of the *PeCER* genes was identified by extracting 1,000-bp upstream sequences from all the 34 *PeCER* genes, and was analyzed using the PTRM online database. The results showed that there were 2402 TFs from numerous TF families including ERF, TCP, MYB, NACWRKY, BBR-BPC and bHLH, which were involved in the regulation of the 34 *PeCER* genes (Supplementary Table S13). Among all the predicted TFs, the most abundant members belonged to the ERF family (1,122), followed by the BBR-BPC (221), TCP (121), MYB (117), and NAC (101) TF families (Figure 7A). The least-targeted TF family members belonged to ARR-B (2), EIL (2), VOZ (2), S1Fa-like (1), and NF-YB (1) (Figure 7A). All the *PeCER* genes were targeted by numerous TFs families members such as *PeCER*33 that interacts with 108, *PeCER*14 with 83, *PeCER*15 with 81, and *PeCER*27 with 56 TF families. In addition, the *PeCER33* gene was the one targeted by most TFs (762), followed by *PeCER15* (267). Among the targeted TF families, the ERF family was dominated by 680 and 216 members in *PeCER33* and *PeCER15* genes (Figure 7B). The TF regulatory network of all the 34 *PeCER* genes are shown in Figure 7A, and the networks of the top five highly targeted *PeCER* genes are shown in Figure 7B, respectively. Fatty acids and wax biosynthesis-related TFs belonging to different TF families were also predicted in this study including ERF, AP2, bHLH, and MYB. Different TFs involved in plant growth and development including TCP, bHLH, BBR-BPC, WRKY, LBD, and AP2 were also found in the *PeCER* genes. In addition, phytohormone-related TFs were also identified, including ERF and ARF. Interestingly, ERF and AP2 TFs were shown to be universally distributed in most of the *PeCER* genes (Figure 7, Supplementary Table S13).

### GO and KEGG Enrichment Analyses of *PeCER* Genes

**Figure 8:**
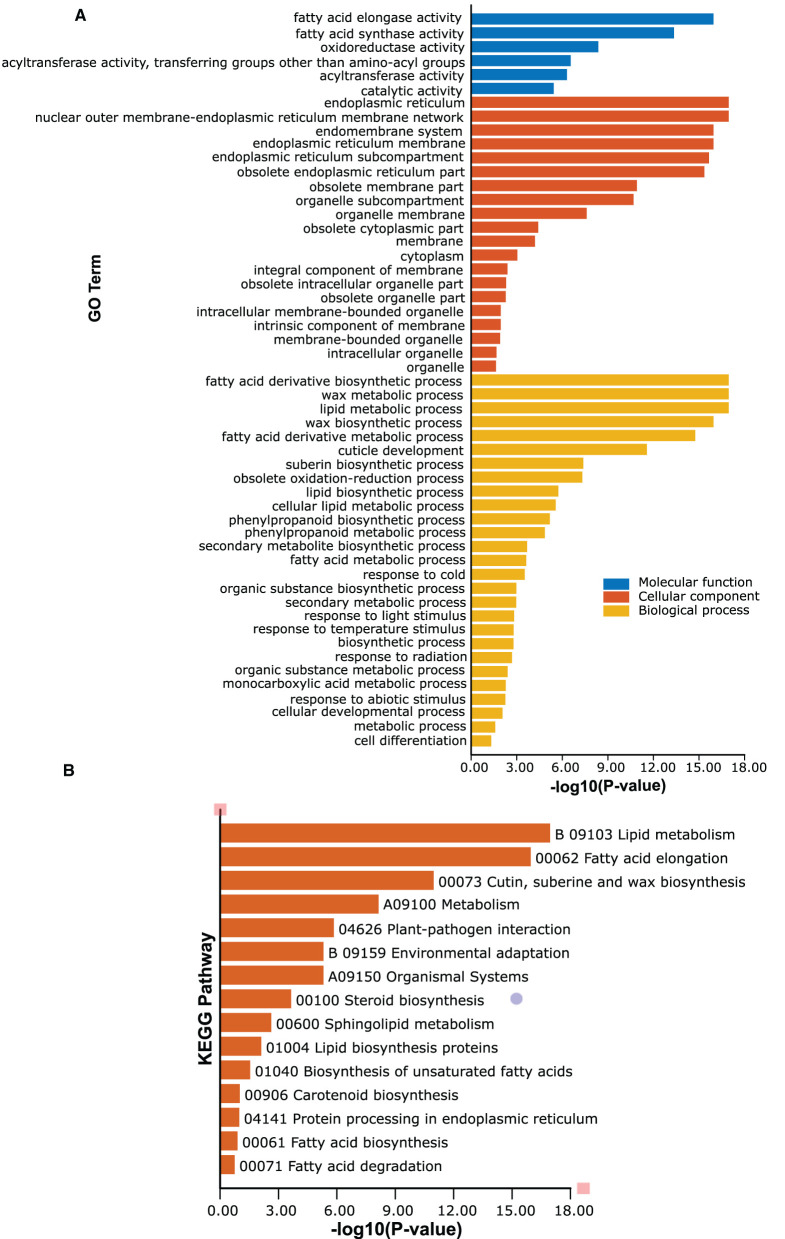
Gene Ontology (GO) and Kyoto Encyclopedia of Genes and Genomes (KEGG) enrichment analyses of *PeCER*s genes. **(A)** Highly enriched GO terms in *PeCER* genes. **(B)** Highly enriched KEGG pathways in *PeCER* genes. Further detailed annotation results and numerous significantly enriched terms of MF, CC, BP, and KEGG pathways can be found in Supplementary Tables S14A, S15.

GO and KEGG function annotation and enrichment analyses of passion fruit *PeCER* genes were performed. The GO function annotation and enrichment analysis was classified into the molecular function (MF), cellular component (CC), and biological process (BP) classes. The detailed annotation results and highly enriched terms of MF, CC, and BP have been provided in Supplementary Tables S14A,B. The results showed that the highest 200 terms were found in the GO-BP class, followed by 90 terms in GO-MF and at least 29 terms in the GO-CC class (Supplementary Table S14A). In terms of high enrichment analysis, there were 27 highly enriched terms in the GO-BP class inducing metabolic process (GO:0008152), lipid metabolic process (GO:0006629), organic substance biosynthetic process (GO:1901576), fatty acid derivative biosynthetic process (GO:1901570), wax metabolic process (GO:0010166), wax biosynthetic process (GO:0010025), cuticle development (GO:0042335), response to cold (GO:0009409), response to temperature stimulus (GO:0009266), and response to light stimulus (GO:0009416). Six terms were highly enriched in the GO-MF class including catalytic activity (GO:0003824), oxidoreductase activity (GO:0016491), fatty acid elongase activity (GO:0009922), fatty acid synthase activity (GO:0004312), and acyltransferase activity (GO:0016746-47). The GO-CC enrichment results exhibited 20 highly enriched terms including endoplasmic reticulum (GO:0005783), endomembrane system (GO:0012505), obsolete cytoplasmic part (GO:0044444), membrane-bounded organelle (GO:0043227), and organelle membrane (GO:0031090) (Figure 8A, Supplementary Table S14B).

The KEGG pathway analysis revealed that there were 31 KEGG pathways predicted in the 34 *PeCER* genes; among them, the highly enriched pathways are shown in Figure 8B including metabolism (A09100), lipid metabolism (B09103), fatty acid elongation (00062), cutin, suberine, and wax biosynthesis (00073), plant-pathogen interaction (04626), environmental adaptation (B09159), and organismal systems (A09150) (Figure 8B, Supplementary Table S15). Overall, the GO and KEGG enrichment analyses show that the *PeCER* genes might play an important role in several biological, molecular, and cellular processes such as plant metabolism, fatty acid and wax biosynthesis as well as in response to biotic and abiotic stress.

### Subcellular Localization of PeCER32 Protein

**Figure 9:**
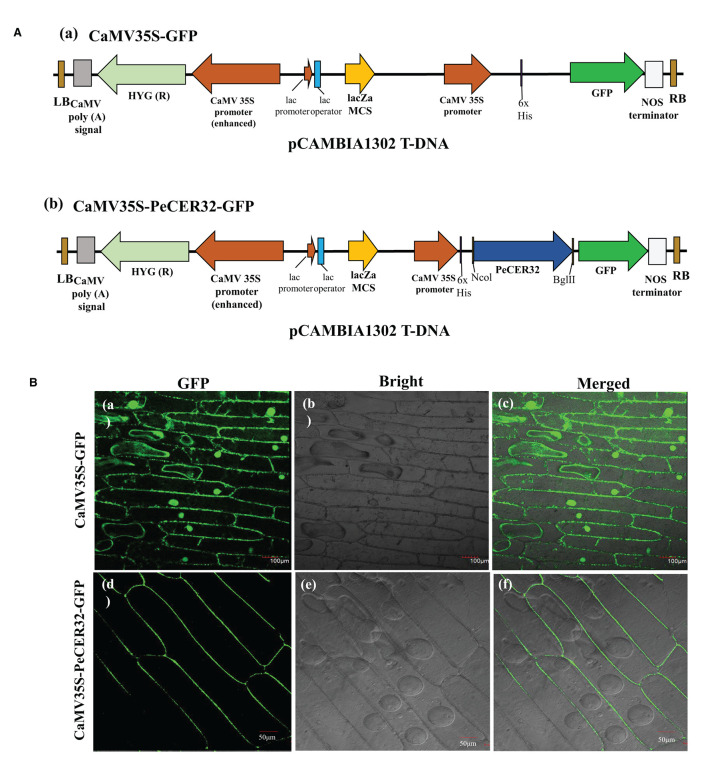
Subcellular localization of GFP-fused PeCER32 protein based on transient expression assays in onion epidermal cells. **(A)** (a) Structure of CaMV35S-GFP as empty vector. (b) Structure of CaMV35S-*PeCER32*-GFP as construct. **(B)** (a–c) Onion cells transformed with CaMV35S-GFP as control (scale bar = 100 μm). (d–f) Onion cells transformed with CaMV35S-*PeCER*32-GFP (scale bar = 50 μm). (a,d) GFP signals visualized under dark field. (b,e) Onion epidermal cells under bright light. (c,f) represent merging of (a,b,d,e).

Most of the PeCER proteins were hypothetically predicted to be localized to the plasma membrane (Table 1). The validation of predicted subcellular localization was performed by selecting a PeCER32 protein, which were strongly evolved in various predicted functions including metabolism, organ-specific, organ developmental, and different stress responses. A transient expression assay was carried out by transformation with the CaMV35S-PeCER32-GFP fusion construct (Figure 9Aa) and empty vector CaMV35S-GFP (Figure 9Ab) into onion epidermal cells with the agroinfiltration method. The results showed that GFP signals were highly expressed in the plasma membrane (Figures 9Bd–f). The empty vector CaMV35S-GFP was used as control, and the results showed a dispersed pattern of GFPs throughout the whole cell (Figures 9Ba–c). The subcellular localization results of PeCER32 protein were consistent with the most preferential prediction.

### Expression Profiles of *PeCER* Genes in Different Fruit Development Stages

**Figure 10:**
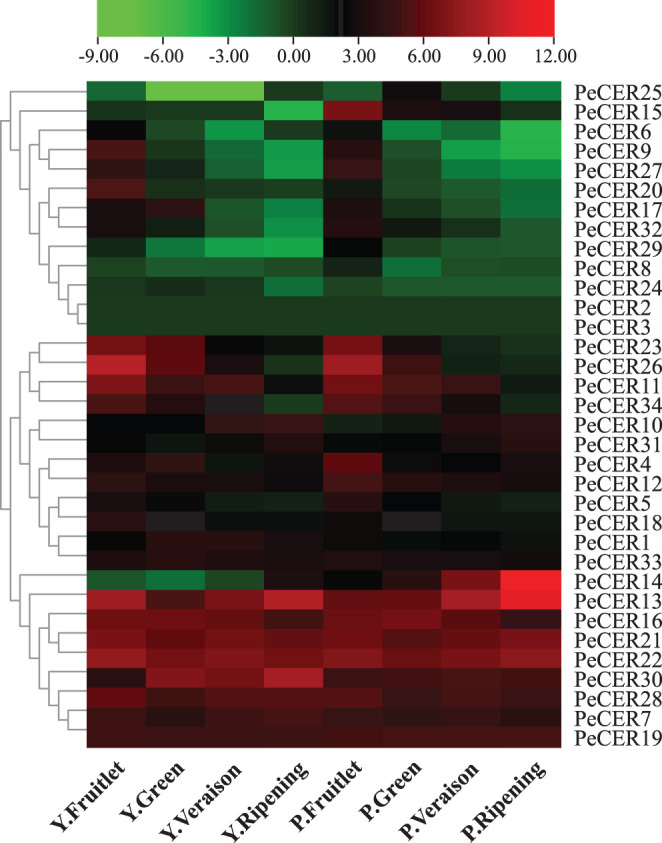
Expression profiles of *PeCER* genes in pulp tissue of yellow and purple passion fruit cultivars in four fruit development stages. Y and L represent the yellow (Y) and purple (P) passion fruit cultivars. FPKM values were transformed by log2, and heatmap was constructed with the TBTools software. The red color shows the highest and the blue color shows the lowest expression levels in expression bar.

The expression profiles of the 34 *PeCER* genes in pulp tissues of yellow and purple passion fruit cultivars were further evaluated based on FPKM expression levels in four fruit development stages. The FPKM expression values have been provided in Supplementary Table S16. The FPKM values were transformed to log^2^FC, and a heatmap was generated with the TBtools software. The expression profiles of *PeCER* genes varied between both cultivars and developmental stages. Taken together, the results showed that 32 (94%) of the 34 (100%) *PeCER* genes were expressed during fruit development and ripening in both cultivars (Figure 10, Supplementary Table S16).

*PeCER2* and *PeCER3* were not expressed in all the tissues, indicating that they might not be involved in fruit development. In the yellow passion fruit cultivar, 32 (94%), 31 (91%), 31 (91%), and 30 (88%) *PeCER* genes were expressed in the fruitlet, green, veraison, and ripening stages. Among the expressed genes, 17 (53%), 16 (51%), 11 (35%), and 10 (33%) *PeCER* genes were highly expressed (FPKM > 10) in the fruit development and ripening stages. In addition, 9 genes (*PeCER*7, *PeCER*13, *PeCER*16, *PeCER*19, *PeCER*21, *PeCER*22, *PeCER*28, *PeCER*29, and *PeCER*30) showed an increased expression pattern (FPKM = 13.97 to 440.13) throughout the fruit development and ripening stages. However, another 9 genes (*PeCER*9, *PeCER*11, *PeCER*12, *PeCER*18, *PeCER*20, *PeCER*23, *PeCER*26, *PeCER*27, and *PeCER*34) showed decreased expression level (FPKM = 489.71 to 0.07) from fruit development to ripening stages (Figure 10, Supplementary Table S16).

In the purple passion fruit cultivar, overall 32 (94%) *PeCER* genes were expressed in the fruitlet, green, veraison, and ripening stages. Among the 32 expressed genes, 19 (59%), 13 (41%), 11 (34%), and 11 (34%) *PeCER* genes exhibited high expression levels (FPKM > 10) in all tested stages,. Furthermore, 8 genes (*PeCER*7, *PeCER*13, *PeCER*16, *PeCER*19, *PeCER*21, *PeCER*22, *PeCER*28, and *PeCER*30) showed constantly high expressions (FPKM = 14.59 to 1575.51) through out all the tested stages, whereas 10 genes (*PeCER*4, *PeCER*5, *PeCER*11, *PeCER*12, *PeCER*15, *PeCER*23, *PeCER*26, *PeCER*27, *PeCER*32, and *PeCER*34) exhibited reduced expression levels (FPKM = 271.84 to 0.11) during fruit development to ripening stages. Comparing the *PeCER* expression profiles of both cultivars in fruit development and ripening stages, the results showed that 7 genes (*PeCER*7, *PeCER*13, *PeCER*16, *PeCER*21, *PeCER*22, *PeCER*28, and *PeCER*30) were found to have similar expression between both cultivars in all the tested stages (FPKM > 10). Overall, the purple cultivar had higher expression (FPKM = 3123.21, *PeCER14*) than the yellow (FPKM = 489.7, *PeCER26*). The highest expression level in the purple cultivar was observed during the stage of ripening (FPKM = 3123.22), followed by the veraison (FPKM = 321.01) > fruitlet > (FPKM = 99.3) and green (FPKM = 94.78) stages. In the yellow cultivar, the highest expression patterns were observed in the stage of fruitlet (FPKM = 489.70), followed by ripening (FPKM = 440.13) > veraison (FPKM = 127.38) and green stage (FPKM = 89.90) (Figure 10, Supplementary Table S16). These FPKM expression-based findings suggest that the *PeCER* genes may have significant roles in fruit development and ripening in the yellow and purple passion fruit cultivars, and further research is still needed.

### Expression Pattern of *PeCER* Genes in Different Passion Fruit Tissues

**Figure 11:**
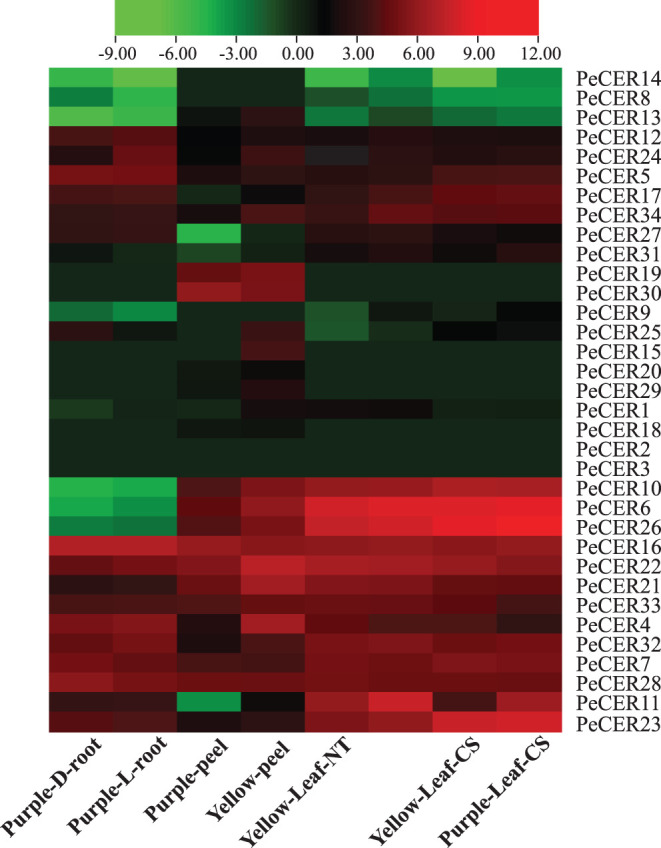
Expression profiles of *PeCER* genes in roots, peels, and leaves of yellow and purple passion fruit cultivars under different conditions. L and D represent the samples from limestone (L) and sandy dolomite (D) rocky desertification areas. NT and CS indicate the normal temperature (NT) and chilling stress (CS) conditions. FPKM values were transformed by log2, and heatmap was constructed with the TBTools software. The red color shows the highest and the blue color shows the lowest expression levels in expression bar.

The *PeCER* gene expression patterns in roots of the purple cultivar, and leaves and peel tissues of the yellow and purple cultivars were evaluated based on FPKM values. The FPKM expression values have been provided in Supplementary Table S17. The FPKM values were transformed to log^2^FC, and a heatmap was constructed with TBtools (Figure 11, Supplementary Table S17). Among all the 34 *PeCER* genes, 26 (76.47%) genes were expressed in purple L and D roots; 28 (82%) genes were expressed in yellow and purple leaves under NT and CS conditions; whereas, 29 (85%) genes were expressed in yellow and purple peel tissues, respectively. Among the expressed *PeCER* genes in the L and D roots, 12 (46%) and 11 (42%) genes were highly expressed (FPKM > 10). Comparing the expression levels of *PeCER* genes in L and D roots, the expression levels of root genes in L conditions (FPKM = 120.04, *PeCER16*) had the higher expression than those in D conditions (FPKM = 119.09, *PeCER16*). Among the expressed *PeCER* genes in purple and yellow peels, 11 (39%) and 16 (55%) *PeCER* genes showed highest expressions (FPKM > 10) in the yellow and purple peels. The purple peels showed highest expression level in *PeCER*22 (FPKM = 144.42) compared with yellow *PeCER*30 (FPKM = 58) (Figure 11, Supplementary Table S17).

The expression pattern in leaf tissues of the two cultivars under CS and NT conditions showed that 15 (58%), 13 (50%), 15 (58%), and 16 (61%) *PeCER* genes were highly expressed (FPKM > 10) in purple leaf-NT, yellow leaf-NT, purple leaf-CS, and yellow leaf-CS conditions. Comparing the leaf expressions of the two cultivars under CS and NT conditions, the highest expression was found in purple leaf-CS (FPKM = 647.01, *PeCER26*), followed by yellow leaf-CS (FPKM = 394.1, *PeCER26*), purple leaf-NT (FPKM = 303.78, *PeCER6*), and yellow leaf-NT (FPKM = 266.04, *PeCER6*). Taken together, the purple cultivar showed the highest expression in all the tested tissues such as purple leaf-CS > yellow leaf-CS > purple leaf-NT > yellow leaf-NT followed by purple L-root > purple D-root, and the lowest expression was found in the yellow peel (Figure 11, Supplementary Table S17). *PeCER2* and *PeCER3* were not expressed in all the tested tissues, indicating that they might not be involved in fruit or plant development, whereas *PeCER6* and *PeCER16* showed the highest expression, indicating that these genes might have important roles for specific functions in all the tissues, but further research is needed. These genotype-based tissue expression patterns provide ideas for further studies on plant and fruit development of the *PeCER* gene family in passion fruit.

### Expression Profiles of *PeCER* Genes Under Drought Stress Conditions

**Figure 12:**
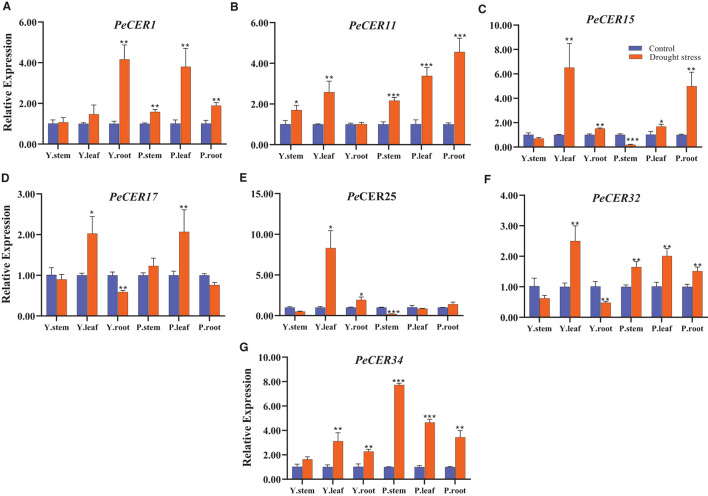
Relative expressions of *PeCER* genes **(A–G)** in stem, root, and leaf tissues of yellow and purple passion fruit plants under control and drought stress conditions. Relative gene expression levels were calculated using 2^−Δ*Δct*^. Plants with regular watering were taken as control. Vertical bars represent means ± SD (*n* = 3). *, **, and *** show significance at *p* ≤ 0.05, *p* ≤ 0.01, and *p* ≤ 0.001, respectively, under control and drought stress condition according to Students *t*-test. Y represents yellow passion fruit, and P represents purple passion fruit.

*CER* genes have been reported to play important roles in drought stress conditions; to evaluate the expression profile of the *PeCER* genes under drought stress conditions, seven passion fruit *PeCER* genes were selected based on their significantly different FPKM expressions, *cis*-elements, and GO terms to participate in various stress responses and other functions. A qRT-PCR expression analysis was performed on the seven selected *PeCER* genes (Figure 12). Overall, all the seven *PeCER* genes exhibited diverse expression patterns in stems, leaves, and root tissues of the yellow and purple passion fruit cultivars under drought stress conditions. The results showed that the expression levels of most of the genes were upregulated in cultivars subjected to drought stress conditions as compared to the controls (Figure 12).

Overall all the seven *PeCER* genes were highly upregulated in leaf tissues compared with stem and root tissues (Figure 12). The *PeCER*25 gene showed the highest expression level (8.29-fold) in the leaf tissue of the yellow cultivar (Figure 12E), followed by *PeCER*34 (7.73-fold) in the stem tissue of the purple cultivar (Figure 12G) under drought conditions compared with the controls. *PeCER*15 showed higher expression levels (6.51-fold) in yellow cultivar leaves and purple cultivar root tissues (4.99-fold), while downregulated in both cultivar stem tissues under drought stress conditions compared to the control (Figure 12C). The *PeCER*17 gene showed consistent expression levels in yellow and purple leaves and root tissues among genotypes (Figure 12D). In the comparison of expression levels in tissues of the two genotypes, the stem tissues of the purple cultivar showed the highest expression level (1.7-fold) (Figure 12B), whereas the leaves (8.29-fold) (Figure 12E) and root (4.16-fold) (Figure 12A) tissues of the yellow cultivar showed highest expressions under drought stress conditions compared to the controls. These findings indicated the importance of *PeCER* genes under drought stress conditions and provide a foundation for further functional studies.

### Expression Profiles of *PeCER* Genes Under Biotic Stress Conditions

**Figure 13:**
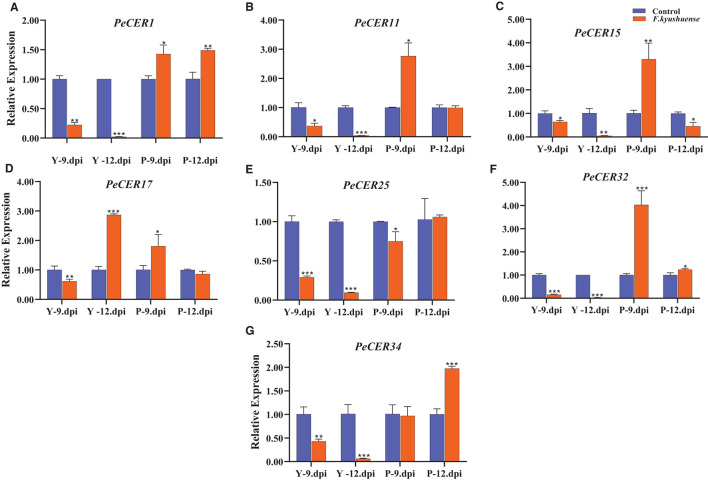
qRT-PCR expression profiles of *PeCER* genes **(A–G)** in peel tissues of yellow and purple passion fruit cultivars under *F. kyushuense* biotic stress and control conditions. Relative gene expression levels were calculated using 2^−Δ*Δct*^. Vertical bars represents means ± SD (*n* = 3). *, **, and *** show significance at *p* ≤ 0.05, *p* ≤ 0.01, and *p* ≤ 0.001, respectively, under control and biotic stress conditions according to Students *t*-test. Y, yellow passion fruit; P, purple passion fruit; dpi, days post inoculation.

*CER* genes have been reported to play important roles under biotic stress conditions, and the qRT-PCR expression profiles of the seven selected passion fruit *PeCER* genes under *F. kyushuense* (biotic stress) stress conditions were investigated (Figure 13). The *PeCER* gene expression in peel tissues of the yellow and purple passion fruit cultivars at 9^th^ and 12^th^ days post-inoculation (dpi) of the pathogenic fungus *F. kyushuense* was analyzed by comparing with the controls (Figure 13). Overall, all the *PeCER* genes exhibited different expression levels under biotic stress conditions in the yellow and purple cultivars compared to the controls. *PeCER*32 showed the highest expression (4.03-fold) in purple 9 dpi (Figure 13F).

In comparison between the two cultivars at 9^th^ and 12^th^ dpi, the results showed that the expression levels of all the *PeCER* genes were downregulated under *F. kyushuense* stress conditions in the yellow cultivar except for the *PeCER*17 gene and were upregulated in the purple cultivar compared to the controls (Figure 13). The expression of the *PeCER*17 gene was upregulated in yellow (2.87-fold) 12 dpi compared with the control (Figure 13D). The *PeCER*1, *PeCER*11, *PeCER*15, *PeCER*17, and *PeCER*32 genes were highly upregulated (1.42, 2.76, 3.3, 1.80, and 4.03 folds) in the purple cultivars at 9^th^ dpi compared to the controls (Figure 13). However, the *PeCER*15 and *PeCER*17 genes at 12^th^ dpi were downregulated in the purple cultivar compared with the controls (Figures 13C,D). Altogether, the results show that the *PeCER* genes in the passion fruit purple cultivars presented high expressions in cultivars at 9^th^ and 12^th^ dpi samples compared with the yellow cultivar (Figure 13). These results suggest that genes with significantly higher expression levels including *PeCER1, PeCER*11, *PeCER*15, *PeCER*17, and *PeCER*32 may have important roles, and the purple cultivar with highly upregulated expressions under biotic stress provides basic information for further studies on the genetic improvement in passion fruit (Figure 13).

### Validation of FPKM Expression Data by qRT-PCR

**Figure 14:**
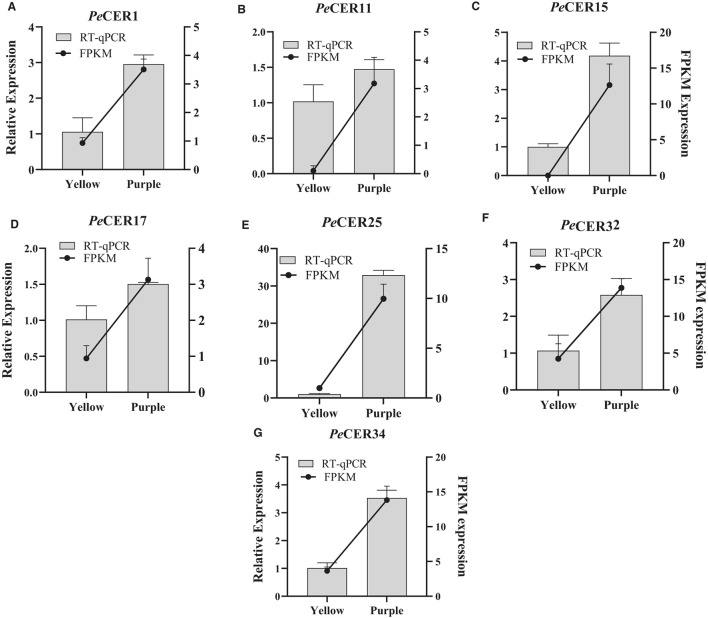
Validation of FPKM expression data by qRT-PCR. **(A–G)** Histograms show the qPCR results of seven PeCER genes in peel tissues of yellow and purple passion fruit cultivars at the ripening stage. Black line charts show the FPKM values of PeCER genes, and vertical gray bars show the qPCR results (2^−ΔΔct^) and represent mean ± SD. The left Y-axis represents the qPCR relative expression levels, and the right Y-axis indicates the FPKM expression values.

According to FPKM expression values, the *PeCER1, PeCER11, PeCER17, PeCER25, PeCER32*, and *PeCER34* genes were significantly expressed in most of the roots, leaves, pulp, and peel tissues, especially in the purple cultivar compared to yellow (Figures 12, 13). The FPKM expression data were further validated by qRT-PCR analysis using the above-mentioned *PeCER* genes in yellow and purple passion fruit peel tissues. After normalization with *Pe*60S, all the tested *PeCER* genes showed a trend line consistent with the FPKM expression values (Figure 14). These results revealed that FPKM expression values provided an appropriate expression result for all the tested tissues between both passion fruit cultivars.

## Discussion

The cuticle of plants is a hydrophobic layer and is the first barrier between the environment and plants to protect them from biotic and abiotic stresses. It is composed of VLCFAs and their derivatives (Yeats and Rose, 2013). Wax biosynthesis has been described in different plant species including *B. napus* (Wang et al., 2020), *O. sativa* (Ni et al., 2018), *A. thaliana* (Aarts et al., 1995; Hooker et al., 2002; Trenkamp et al., 2004; Rowland et al., 2007; Pascal et al., 2013; Kim et al., 2019; Yang et al., 2020b), *Mangifera indica* (Tafolla-Arellano et al., 2017), *Sichuan kumquat* (Yang et al., 2022), *P. pratensis* (Wang et al., 2021), and *M. domestica* (Zhang et al., 2019a, 2020). Different genes are involved in cuticular wax biosynthesis and have been previously reported including *CER* (Bourdenx et al., 2011), KCS (Wang et al., 2020), KCR (beta-ketoacyl-CoA reductase) (Gan et al., 2016), FAR (fatty acyl-CoA reductase) (Wang et al., 2017), LACS (Schnurr et al., 2004), CUT1 (Millar et al., 1999), and FAE (fatty acid elongase) (Millar and Kunst, 1997). *CER* is one of the main gene families involved in cuticle wax biosynthesis and stress response (Bourdenx et al., 2011). The genome-wide identification of *CER* family genes has been reported in different plant species including *Z. jujuba* Mill (Li et al., 2021), *M. domestica* (Qi et al., 2019a), and *H. annuus* (Ahmad et al., 2021) and has been functionally characterized in *A. thaliana* (Aarts et al., 1995; Hooker et al., 2002; Trenkamp et al., 2004; Rowland et al., 2007; Pascal et al., 2013; Kim et al., 2019; Yang et al., 2020b), *P. pratensis* (Wang et al., 2021), *O. sativa* (Ni et al., 2018), *B. napus* (Wang et al., 2020), and *Brachypodium distachyon* (Wu et al., 2019). Information on *CER* genes in *P. edulis* remained unknown, but the availability of the passion fruit genome (Ma et al., 2021) made it possible studies on genomic levels.

In this study, 34 *PeCER* genes were identified in the passion fruit genome and were unevenly distributed across eight chromosomes, with the largest number of genes located on chromosome 1 (Table 1, Supplementary Figure S1). Ahmad et al. (2021) mentioned that the diversity of genes in the same family on different chromosomes may be due to their involvement in various functions. The 34 identified *PeCER* genes in the passion fruit genome were relatively similar in terms of number to sunflower *CER* (thirty-seven genes) (Ahmad et al., 2021), jujuba *CER* (twenty-nine) (Li et al., 2021), while some species have fewer *CER* genes including apple, which has 10 *CER* genes (Qi et al., 2019a); the difference in gene number may be due to differences in genome size. The phylogenetic tree was constructed among *P. edulis, A. thaliana, M. domestica, H. annus*, and *Z. jujuba* proteins and divided into 7 clades according to sunflower *CER* (Ahmad et al., 2021) (Figure 1A). Liu et al. (2018) pointed out that genes present in the same clade might perform similar functions. Differences in protein functions can be described by identifying conserved motifs during the development of different gene families (Wong et al., 2015). Furthermore, conserved motifs were predicted in PeCER proteins and a diverse motif pattern ranging from 1 to 24 motifs was found, indicating that PeCER proteins have a remarkably conserved protein structure (Figure 1B). Our results are inconsistent with Li et al. (2021), who also found similar motif results in jujuba.

Differences between the number of exons and introns provide an important source for gene family variation and determine the different functions and expressions of genes (Xu et al., 2012). In this study, a quite different number of introns were found in the *PeCER* genes ranging from 0 to 28 introns. The *PeCER*6, *PeCER*8, and *PeCER*9 genes have no introns, and similar results have been reported previously for intron-less genes such as *AtCER6*-2 and *HanCER6*-2 (Ahmad et al., 2021), while *PeCER*13 contained 28 introns (Figure 1C). These results indicate that ancestors of the *PeCER* genes might have gone through several rounds of intron loss and gain during development (Frugoli et al., 1998).

The *cis*-regulatory element analysis of *PeCER* genes revealed that ABRE, CGTCA-motif, GARE-motif, G-Box, Box 4, and ARE *cis*-elements were found in abundance (Figure 2), indicating that *PeCER* might be involved in different plant developmental and stress responses (Wingender et al., 1990; Wang et al., 2011; Xiong et al., 2020). Fujita et al. (2013) demonstrated the important roles of the ABRE element in ABA and stress responses. Kaur et al. (2017) reported the involvement of GARE *cis*-elements in hormone responsiveness. Liu et al. (2016) reported the involvement of W-box and G-box elements in early senescence of rice flag leaf. Gene duplication is a prominent feature of plant genomics that could lead to evolutionary and functional novelties that arise from existing genes (Flagel and Wendel, 2009). The differences in *CER* genes and chromosome numbers among passion fruit, apple, sunflower, jujuba, and *Arabidopsis* species suggest that they have gone through lineage-specific genome duplication. Whole-genome duplication (WGD) consists of tandem and segmental duplications that cause a sudden increase in the size of a genome and entire gene sets by generating two gene copies (Van De Peer et al., 2009). The results of the synteny analysis and calculation of Ka/Ks values in *PeCER* genes showed that the duplication process between the tandem and segmental *PeCER* genes was estimated to be 1.12 to 20.78 mya (Supplementary Table S6), which is consistent with a published report on passion fruit, indicating that passion fruit has gone through WGD twice (12 and 65 mya) (Xia et al., 2021).

Six segmental and two tandem duplicate *CER* genes in the passion fruit genome (Figure 3A, Supplementary Table S6) were identified, which is consistent with Xia et al. (2021) who also found two tandem repeats in the passion fruit TPS-b subfamily. Furthermore, the synteny analysis of *P. edulis CER* genes with *A. thaliana, M. domestica*, and *Z. jujuba CER* genes identified 10 *PeCER* genes as orthologous to 12 *CER* genes from the above species (Figure 3B). The multicollinearity analysis of the *P. edulis CER* genes revealed the highest collinearity between *P. edulis* and *M. domestica* (43 collinear genes). Santos et al. (2014) reported that compared to dicot genomes, the number of repetitive elements between *P. edulis* and *M. domestica* genomes was 42.4% (Figure 4), suggesting that these orthologous may share the same ancestors and retain corresponding functions.

The protein--to-protein interaction network of a particular gene family provides evidence of the relationship between members (Piya et al., 2014). The protein-to-protein interaction results of PeCER proteins showed that seven PeCER proteins were homologous with AtCER1 (Figure 5A). Bernard and Joubès (2013) reported that AtCER1 dynamically contributes to wax biosynthesis and actively responds to biotic and abiotic stresses. Two PeCER proteins showed homology with AtCER3, and it has been reported that AtCER3 interacts with AtCER1 and catalyzes the redox-dependent VLCAs from very long-chain-Acyl-CoAs (Wu et al., 2019). Two PeCER proteins showed homology with AtSTE1 and interact with AtDWF1 and AtDWF5. Silvestro et al. (2013) stated that AtSTE1 (Delta(7)-sterol-C5(6)-desaturase1) is a precursor of growth-promoting brassinosteroids and is involved in sitosterol and campesterol biosynthesis. Choe et al. (2000) stated that AtDWF57-dehydrocholesterol reductase was involved in cholesterol production. Some PeCER proteins showed homology with AtKCS. Batsale et al. (2021) described that AtKCS was involved in the biosynthesis of VLCFAs and essential for cuticular wax and suberine biosynthesis. Two PeCER proteins showed interaction with AtSBH (sphingoid base hydroxylases). Shu et al. (2015) stated that AtSBH is involved in sphingolipid trihydroxy long-chain base (4-hydroxysphinganine) biosynthesis and response to high humidity and temperature stress. The PeCER protein-to-protein interaction results suggest that the homology and interaction with known *Arabidopsis* proteins may have the same functions such as wax biosynthesis, plant development, and stress responses, but further studies are required (Figure 5A).

The protein's secondary structures are the physical arrangement of amino acid sequences and are highly conserved between homologous proteins. The order of amino acids directly affects protein folding, 3D structure, and functions (Ridout et al., 2010). The results of PeCER protein secondary structure and 3D modeling indicate that the alpha helix accounts for the largest percentage of secondary structures (57.2%), followed by random coils (40.95%) and extended strands (25.68%) (Supplementary Table S11), and our results are identical to those of Lian et al. (2020), who also found the largest percentage of secondary structures followed by random coils and extended strands. In terms of 3D modeling, most of the PeCER proteins showed similar 3D structures except for a few of them (Figure 5B), indicating that the identified PeCER proteins were conserved and consistent with motif and gene structure analysis. Ridout et al. (2010) pointed out that protein structures, similar or different, may be due to differences in amino acid sequence size or arrangement.

In recent times, different miRNAs have been identified in numerous species, such as maize (*Zea mays*) (Aravind et al., 2017), cowpea (*Vigna unguiculata*) (Barrera-Figueroa et al., 2011), soybean (*Glycine max*) (Song et al., 2011), peanut (*Arachis hypogaea*) (Zhao et al., 2015), and passion fruit, that are involved in different metabolism, development, and environmental stresses (Paul et al., 2020). In this study, 12 putative miRNAs targeting 16 *PeCER* genes were identified (Figure 6, Supplementary Table S12), and two members of the ped-miR166 family targeted four *PeCER* genes. miR166 has been reported to be involved in drought stress in maize (Aravind et al., 2017) and cowpea (Barrera-Figueroa et al., 2011), seed development in soybean (Song et al., 2011), disease resistance in peanut (Zhao et al., 2015), development of protein metabolic process, shoot apical meristem in passion fruit (Paul et al., 2020), plant growth, development, and stress response in apple (Varkonyi-Gasic et al., 2010).

Two members of the ped-miR171 family targeted two *PeCER* genes, and miR171 has been reported to be involved in development, metabolism, and photosynthesis in grapevine (*Vitis vinifera*) (Han et al., 2014), in coffee (*Coffea Arabica*) (Chaves et al., 2015); stress response in tea plant (*Camellia sinensis*) (Zhang et al., 2014), and development and defense responses in passion fruit (Paul et al., 2020). One member of the ped-miR395 family targeted three *PeCER* genes. miR395 was found to be involved in abiotic stress in wheat (*Triticum aestivum*) (Han et al., 2013), starch metabolism in cassava (*Manihot esculenta*) (Patanun et al., 2013), development and stress response in sorghum (*Sorghum bicolor*) (Katiyar et al., 2012a; Su et al., 2021), and salt stress tolerance in cotton (*Gossypium hirsutum*) (Wang et al., 2013). Two members of the ped-miR399 family targeted three *PeCER* genes and have been stated to be involved in drought stress response in barley (*Hordeum vulgare*), sugarcane (*Saccharum officinarum*) (Zanca et al., 2010), phosphate homeostasis, signaling, transport in apple (Pant et al., 2008), and phosphate deficiencyin sorghum (Katiyar et al., 2012a). These findings proposed that the identified ped-miRNAs may play key roles in combating multiple stresses by modifying the transcriptional level of *CER* genes in passion fruit but requires further studies.

Plant TFs have been reported to be involved in regulation of fatty acids and wax biosynthesis, as well as stress responses under different conditions (Hao et al., 2017). In this study, various TFs targeting the 34 *PeCER* genes were predicted and their regulatory network interactions with the *PeCER* genes were constructed (Figure 7, Supplementary Table S13). The ERF TF family was found to be the most abundant, followed by BBR-BPC, TCP, MYB, NAC, and AP2, indicating their positive or negative roles in wax and fatty acid biosynthesis and different stresses. Go et al. (2014) found that wax biosynthesis in *Arabidopsis* was negatively regulated by AP2/ERF-type TFs. The overexpression of AP2/ERF-type TFs in *Arabidopsis* and tobacco increased the load of wax and drought resistance (Yang et al., 2020a). BBR-BPC TFs response to cytokinin and control flower development, brassinosteroid signaling, size of stem cells, and seed development (Shanks et al., 2018; Theune et al., 2019). TCP TFs regulate plant growth, development, and stress response (Li, 2015; Danisman, 2016; Mir et al., 2022). MYB TFs regulate VLCFA biosynthesis, plant development, metabolism, and response to stresses (Raffaele et al., 2008; Katiyar et al., 2012b; Ambawat et al., 2013). Zhang et al. (2019b) reported that the MYB30 TF played an important role in cuticular wax biosynthesis and resistance to pathogens. NAC TFs play an important role in plant immunity and biotic and abiotic stresses (Nuruzzaman et al., 2013). Our results are consistent with previous reports indicating that the role of TFs in *PeCER* genes may be to regulate plant stress resistance, while the wax regulation analysis requires further investigation.

GO aims to functionally classify genes into distinct terms, which can be further classified into three ontology categories at the genome level: GO-BP, GO-CC, and GO-MF (Ashburner et al., 2000). KEGG bioinformatics resources can be used to obtain genome-level biological functions in pathway maps (Masoudi-Nejad et al., 2007). GO and KEGG annotation and enrichment analyses were also performed on the *PeCER* genes (Figure 8, Supplementary Tables S14, S15). Most of the GO terms were related to organelle membrane, fatty acid synthase, wax and lipid metabolic process, organic substance, and stress response. Highly enriched KEGG pathways among the *PeCER* genes include lipid and fatty acid metabolism, cutin, suberine and wax biosynthesis, and plant-pathogen interaction. Our results are consistent with previous reports (Ni et al., 2016; Qi et al., 2019b), which also found similar GO terms and KEGG pathways related to wax and fatty acid biosynthesis, plant-pathogen interaction, and different stress responses. Cuticular wax plays an important role in plant biotic and abiotic stress tolerance. *CER* genes play an important role in VLCFA and wax biosynthesis (Li et al., 2021). *CER10* is involved in VLCFA biosynthesis (Zheng et al., 2005), and the *CER10* mutant exhibits increase in non-stomatal water loss and tolerance to drought conditions (Ensing et al., 2016). The expression of *AtCER1* and *AtCER3* improved the cuticle wax and reduced water loss under drought stress conditions in tobacco (Cameron et al., 2006). The FPKM expression results showed that the *PeCER* genes were differentially and constitutively expressed in different passion fruit tissues under different conditions (Figures 13, 14).

The FPKM expression results showed that compared with NT leaves, the expression levels of *PeCER* genes were higher in CS leaves, which were consistent with qRT-PCR expression results that the expressions of *PeCER* genes in drought-stressed leaves were higher compared to the control group (Figures 13, 14). Similarly, Wang et al. (2021) also found that the expression of *PpCER1*-2 was higher in drought-stressed leaves than in watered leaves. Wang et al. (2020) also pointed out that the *BnCER1*-2 in brassica-overexpressed plants stimulates the production of alkanes and improves tolerance to drought. *Pp*CER1-2 was constitutively expressed in multiple tissues, with high expression in stems and leaves but very low expression in root tissues (Wang et al., 2021). In rice, *OsCER1* was highly expressed in the tapetum and bicellular pollen cells but lower in vegetative organs, suggesting a role in organ development. Based on the FPKM and qRT-PCR expression results between different tissues and *PeCER* genes, the results showed diverse expressions such as *PeCER*1, *PeCER*11, *PeCER*15, and *PeCER*32 were highly expressed in roots and leaves but not in stem tissues. Similar results were found in Pascal et al. (2013), where *CER26* was highly expressed in leaves but not in stems, *CER2* was expressed in all tissues, and *CER26*-like was highly expressed in flowers but low in all other tissues.

Rowland et al. (2006) also found that *CER4* was involved in the production of *Arabidopsis* epidermal wax and expressed in different plant tissues such as plant stems, roots, flowers, leaves, and siliques. Furthermore, cuticle wax also played an important role in plant defense against bacterial and fungal pathogens. The qRT-PCR expression results of *F. kyushuense* (biotic stress) showed that the *PeCER* genes under biotic stress conditions were upregulated in the purple cultivar compared with the controls, and are consistent with Bourdenx et al. (2011) who demonstrate that *CER1* was involved in alkane biosynthesis and was highly correlated with responses to biotic and abiotic stresses. Wang et al. (2015) also reported that the overexpression of cucumber *CsWAX2* homologous to *CER3* showed a significant increase in resistance to drought and pathogens. The qRT-PCR expression results of *PeCER* genes were also consistent with the predicted TF results that the expression levels of *PeCER* genes containing numerous wax- and VLCFA-related transcription factors (EFR) were upregulated under stress conditions compared to the controls. Overall, these results suggest that the *PeCER* genes showed an important role by positive or negative regulation under stress conditions. In future studies, the identified *PeCER* genes could be used for further functional studies on wax biosynthesis and stress associated with the purpose of genetic improvement of passion fruit.

## Conclusion

In this study, comprehensive analyses were performed, and 34 *PeCER* genes in the passion fruit genome were identified. All the *PeCER* genes were subjected to physio-chemical features, evolutionary relationships, chromosomal mapping, motifs, gene structures, *cis*-regulatory elements, protein-protein interaction, syntenic analysis, TFs regulatory network analysis, GO andKEGGannotation, and putative miRNA prediction analysis. The *PeCER* genes were expanded and subjected to purification selection. The predicted subcellular localization was validated by transient expression assay of the PeCER32 in onion epidermal cells. Different TFs including ERF, TCP, MYB, and NAC were identified, and a TF regulatory network associated with the *PeCER* genes was constructed. Diverse FPKM expression profiles of the *PeCER* genes were found in the roots, peels, leaves, and pulp tissues under different conditions. The qRT-PCR expression results revealed that *PeCER1, PeCER11, PeCER15, PeCER17*, and *PeCER32* were highly upregulated under drought and *F. kyushuense* stress conditions compared to the controls but varied in the yellow and purple passion fruit cultivars. These findings provide preliminary information for future functional studies on the *PeCER* genes for the genetic improvement of passion fruit, and the genes including *PeCER1, PeCER11, PeCER15, PeCER17*, and *PeCER32* that were highly expressed under stress may play important roles in wax biosynthesis and stress response, but further functional studies are needed.

## Data Availability Statement

The original contributions presented in the study are included in the article/Supplementary Materials, further inquiries can be directed to the corresponding authors.

## Author Contributions

FC and HMR: conceptualization and validation. AW, SM, MBA, JL, MI, and BL: methodology. HMR and AA: software. HMR, NS, MAAA, and XY: formal analysis. HMR: data curation and writing and original draft preparation. FC, SSS, and RO: review and editing. FC: supervision. ZL and FC: funding acquisition. All the authors have read and agreed to the published version of the manuscript.

## Funding

FC research project was funded by the Department of Science and Technology, Fujian Province, China (2020N0004 and 2020S0056) and the Engineering Research Center of Fujian Province University (G2-KF2006). ZL research project was funded by Enterprise Technology Development (Contract: 2020-3501-04-001995).

## Conflict of Interest

The authors declare that the research was conducted in the absence of any commercial or financial relationships that could be construed as a potential conflict of interest.

## Publisher's Note

All claims expressed in this article are solely those of the authors and do not necessarily represent those of their affiliated organizations, or those of the publisher, the editors and the reviewers. Any product that may be evaluated in this article, or claim that may be made by its manufacturer, is not guaranteed or endorsed by the publisher.
