# Supplementary material for: Comprehensive Genome-Wide Identification and Expression Profiling of Eceriferum (CER) Gene Family in Passion Fruit (Passiflora edulis) Under Fusarium kyushuense and Drought Stress Conditions
Source: Front Plant Sci. 2022 Jun 27;13:898307. doi: 10.3389/fpls.2022.898307 (PMC9272567; doi:10.3389/fpls.2022.898307)

**Supplementary Figure S1. *PeCER* genes position on passion fruit chromosomes.** The scale represents the 300 MB chromosomal distance and the genes are represented in red color.


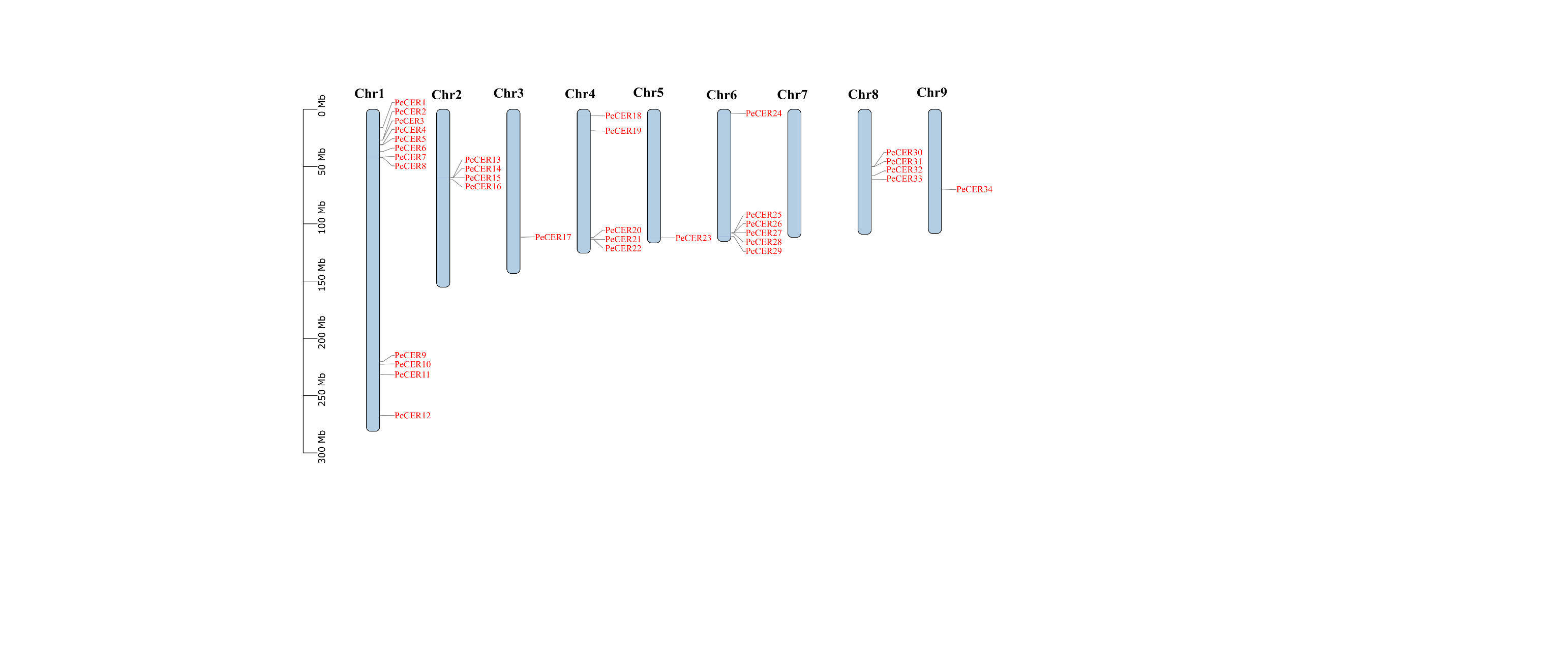

Supplement: Supplementary file 1 [file Data_Sheet_1.ZIP › Supplementary Materials/Supplementary Figure S1.docx]
