# Supplementary material for: Comprehensive Genome-Wide Identification and Expression Profiling of Eceriferum (CER) Gene Family in Passion Fruit (Passiflora edulis) Under Fusarium kyushuense and Drought Stress Conditions
Source: Front Plant Sci. 2022 Jun 27;13:898307. doi: 10.3389/fpls.2022.898307 (PMC9272567; doi:10.3389/fpls.2022.898307)
Supplement: Supplementary file 1 [file Data_Sheet_1.ZIP › Supplementary Materials/Supplementary Table S1.docx]

| **Supplementary Table S1. Primer sequences of *PeCER* genes used in qRT-PCR.** | | |
| --- | --- | --- |
| **Gene** | **Forward primer (5' -3')** | **Reverse primer (5' -3')** |
| ***PeCER*1** | TGGCTTGCCTACATTGCGTA | TGTGATGGGTGGCATGAAGA |
| ***PeCER*11** | TGCTGCTCCATATGCACACT | GCAAAGCGATCCACAACCAA |
| ***PeCER*15** | CTAGCAGCGGCAATTGTACT | TCCTGGATGTTCTTCTGGCA |
| ***PeCER*17** | AGGCCCTCGGTTTTTCCAAA | TGGCCCATTCTCACAGACAT |
| ***PeCER*25** | AGCACGAGAACGAGAAGCTT | ATGTCGTTCCTTTTGGGGCA |
| ***PeCER*32** | CGTCACCCCTCTCCAATGTC | TTTGAAGGTCACCGACAGGG |
| ***PeCER*34** | ACACCGGTTCCTATTCCACA | TGCAGCTGGAGGGAAAACAA |
| ***Pe60S*** | AGGTGGGTAACAGGATTATC | TGGCTGTCTTTTGGTGCTG |
