# Supplementary material for: Comprehensive Genome-Wide Identification and Expression Profiling of Eceriferum (CER) Gene Family in Passion Fruit (Passiflora edulis) Under Fusarium kyushuense and Drought Stress Conditions
Source: Front Plant Sci. 2022 Jun 27;13:898307. doi: 10.3389/fpls.2022.898307 (PMC9272567; doi:10.3389/fpls.2022.898307)
Supplement: Supplementary file 1 [file Data_Sheet_1.ZIP › Supplementary Materials/Supplementary Table S11.docx]

| **Supplementary Table S11. The secondary structure statistics analysis of PeCER proteins.** | | | | |
| --- | --- | --- | --- | --- |
| **Gene name** | **Alpha helix (%)** | **Extended strand (%)** | **Beta turn (%)** | **Random coil (%)** |
| ***PeCER*1** | 37.40 | 19.92 | 6.50 | 36.18 |
| ***PeCER*2** | 39.15 | 18.86 | 7.83 | 34.16 |
| ***PeCER*3** | 38.65 | 23.40 | 5.67 | 32.27 |
| ***PeCER*4** | 45.49 | 15.29 | 4.51 | 34.71 |
| ***PeCER*5** | 49.81 | 12.06 | 3.11 | 35.02 |
| ***PeCER*6** | 47.38 | 13.91 | 5.24 | 33.47 |
| ***PeCER*7** | 50.89 | 12.64 | 4.78 | 31.69 |
| ***PeCER*8** | 45.18 | 15.79 | 4.82 | 34.21 |
| ***PeCER*9** | 48.39 | 14.11 | 5.04 | 32.46 |
| ***PeCER*10** | 45.51 | 13.19 | 5.54 | 35.76 |
| ***PeCER*11** | 48.97 | 10.27 | 1.37 | 39.38 |
| ***PeCER*12** | 40.00 | 19.35 | 4.84 | 35.81 |
| ***PeCER*13** | 31.96 | 24.91 | 8.27 | 34.87 |
| ***PeCER*14** | 39.09 | 19.29 | 5.46 | 36.17 |
| ***PeCER*15** | 34.43 | 25.68 | 9.29 | 30.60 |
| ***PeCER*16** | 32.74 | 19.65 | 6.73 | 40.88 |
| ***PeCER*17** | 42.80 | 14.83 | 2.54 | 39.83 |
| ***PeCER*18** | 42.53 | 15.55 | 6.40 | 35.52 |
| ***PeCER*19** | 57.20 | 12.31 | 5.30 | 25.19 |
| ***PeCER*20** | 46.87 | 15.53 | 5.61 | 31.99 |
| ***PeCER*21** | 40.00 | 18.33 | 4.76 | 36.90 |
| ***PeCER*22** | 35.71 | 18.75 | 8.04 | 37.50 |
| ***PeCER*23** | 39.41 | 19.11 | 7.11 | 34.37 |
| ***PeCER*24** | 38.47 | 15.41 | 5.17 | 40.95 |
| ***PeCER*25** | 41.17 | 17.82 | 5.52 | 35.49 |
| ***PeCER*26** | 41.13 | 20.11 | 5.30 | 33.46 |
| ***PeCER*27** | 46.97 | 15.26 | 5.09 | 32.68 |
| ***PeCER*28** | 53.76 | 12.13 | 1.95 | 32.15 |
| ***PeCER*29** | 44.64 | 14.48 | 4.05 | 36.83 |
| ***PeCER*30** | 43.09 | 13.41 | 5.28 | 38.21 |
| ***PeCER*31** | 45.40 | 13.37 | 5.01 | 36.21 |
| ***PeCER*32** | 33.23 | 25.48 | 3.23 | 38.06 |
| ***PeCER*33** | 53.88 | 11.43 | 4.90 | 29.80 |
| ***PeCER*34** | 41.95 | 15.68 | 2.54 | 39.83 |
