# Supplementary material for: Comprehensive Genome-Wide Identification and Expression Profiling of Eceriferum (CER) Gene Family in Passion Fruit (Passiflora edulis) Under Fusarium kyushuense and Drought Stress Conditions
Source: Front Plant Sci. 2022 Jun 27;13:898307. doi: 10.3389/fpls.2022.898307 (PMC9272567; doi:10.3389/fpls.2022.898307)
Supplement: Supplementary file 1 [file Data_Sheet_1.ZIP › Supplementary Materials/Supplementary Table S4.docx]

| **Supplementary Table S4.** Putative motifs identified from PeCER proteins using MEME. The sequence logos were generated using WebLogo. | | |
| --- | --- | --- |
| **Motif No** | **Sequence logo** | **E-value** |
| **1** | 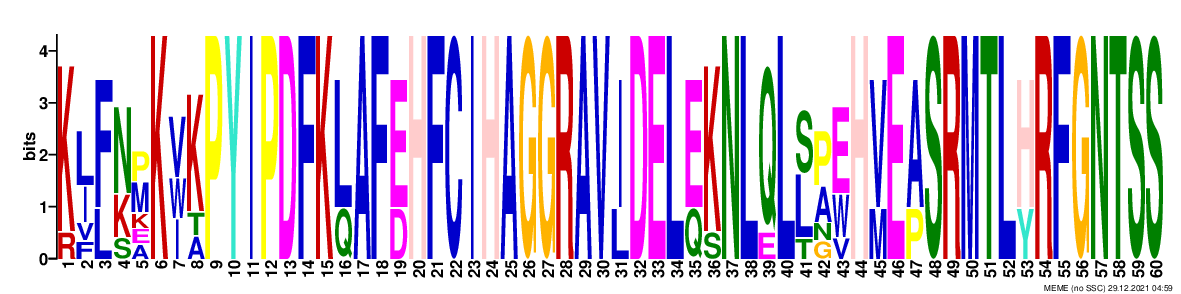  KLFNAKVKPYIPDFKLAFDHFCIHAGGRAVIDELEKNLQLLPEHVEASRMTLHRFGNTSS | **3.5e-249** |
| 2 | 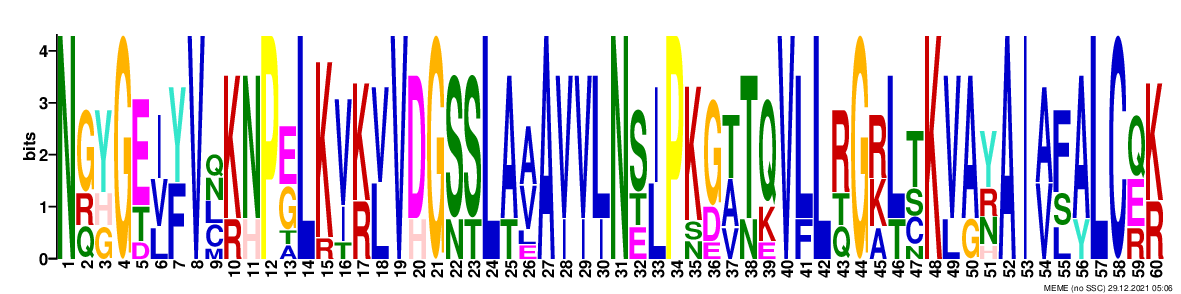  NGYGEIYVLKNPELKVKVVDGSSLAAAVVLNSJPKGTTQVLLRGRLTKVAYAIAFALCZK | **2.3e-242** |
| **3** | 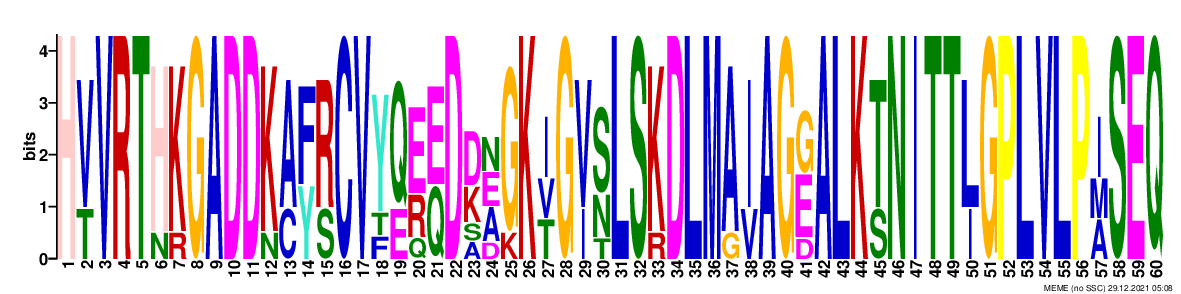HVVRTHKGADDKAFRCVYQEZDDEGKIGVSLSKDLMAIAGEALKTNITTLGPLVLPISEQ | **2.0e-234** |
| **4** | 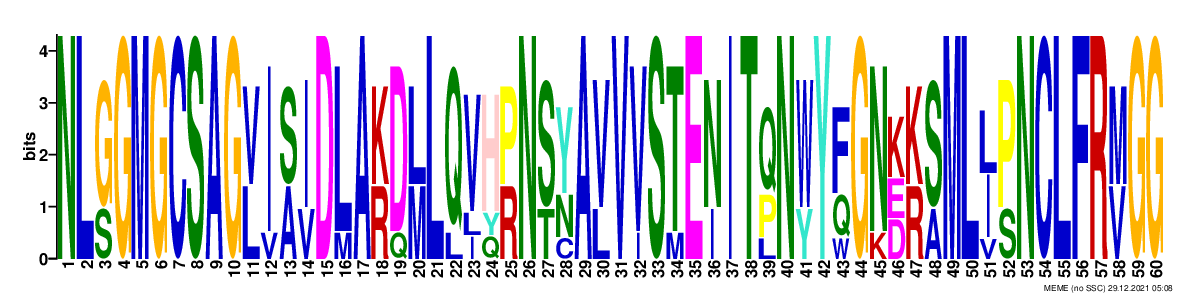NLGGMGCSAGVISIDLAKDLLQVHPNSYAVVVSTENITQNWYFGNEKSMLJPNCLFRMGG | **1.3e-232** |
| **5** | 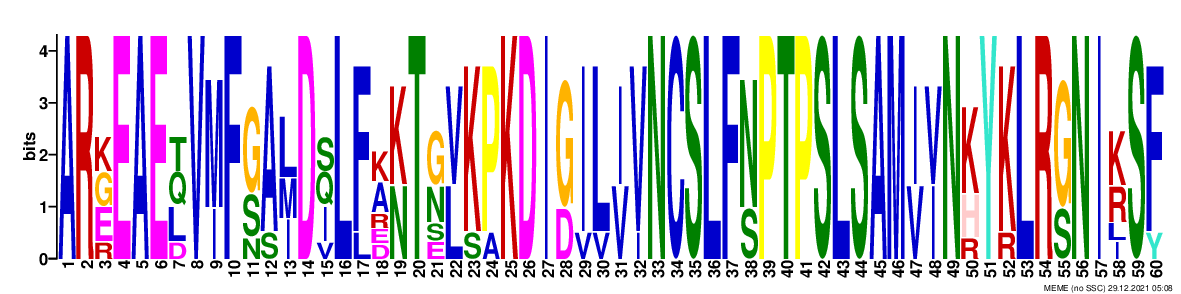ARKEAETVMFGAJDILFKKTGVKPKDIGILIVNCSLFNPTPSLSAMIVNKYKLRGNIKSF | **1.3e-210** |
| **6** | 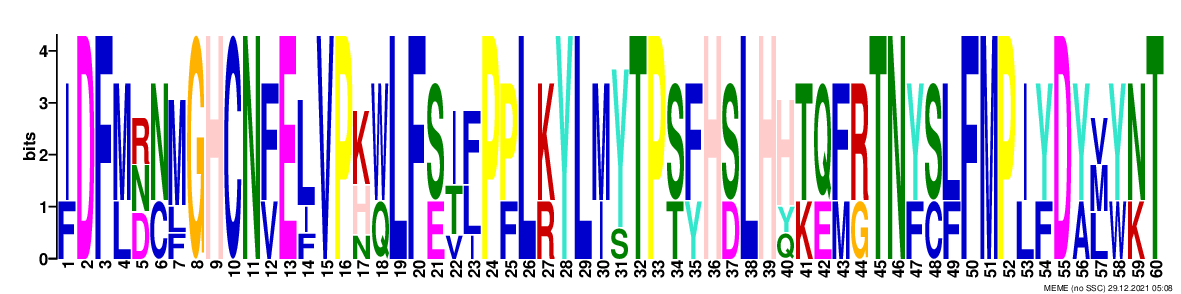IDFMDNMGHCNFELVPKWLFSIFPPLKYLMYTPSFHSLHHTQFRTNYSLFMPIYDYLYNT | **1.4e-180** |
| **7** | 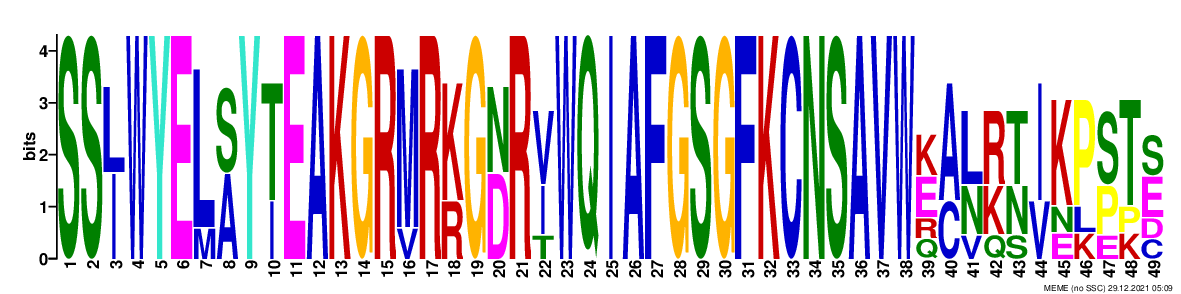SSJWYELAYTEAKGRMRKGBRVWQIAFGSGFKCNSAVWKALRTIKPPTE | **4.5e-151** |
| **8** | 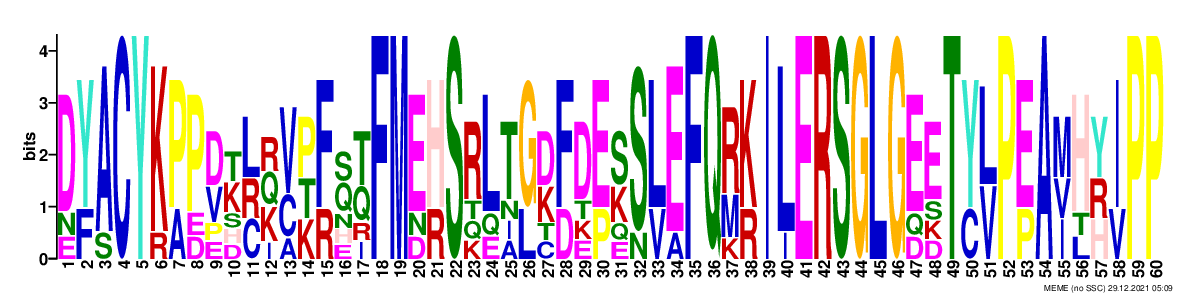DYACYKPPDKLKVPFQTFMEHSRLTGDFDESSLEFQRKILERSGLGEETYLPEAMHYIPP | **2.0e-172** |
| **9** | 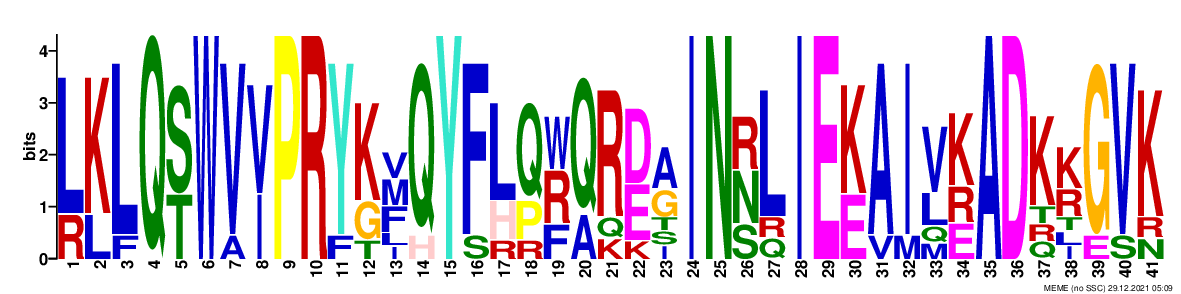LKLQSWVVPRYKVQYFLQWQRDAINNLIEKAIVKADKKGVK | **2.1e-143** |
| **10** | 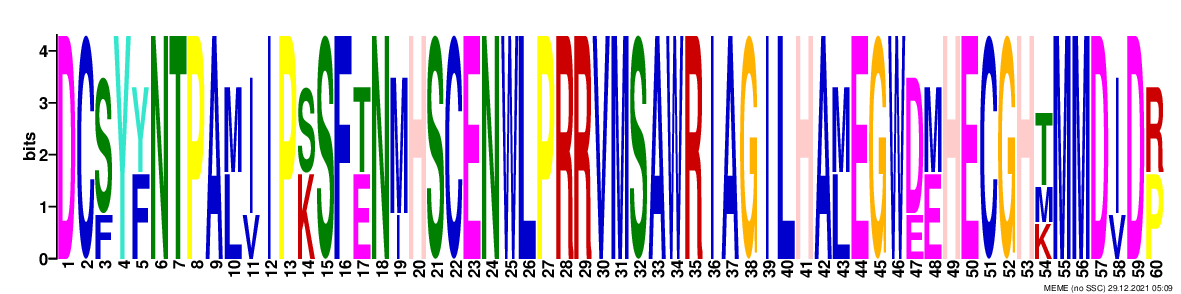DCSYYNTPALIIPKSFENMHSCENWLPRRVMSAWRIAGILHALEGWDEHECGHTMMDIDP | **1.4e-132** |
| **11** | 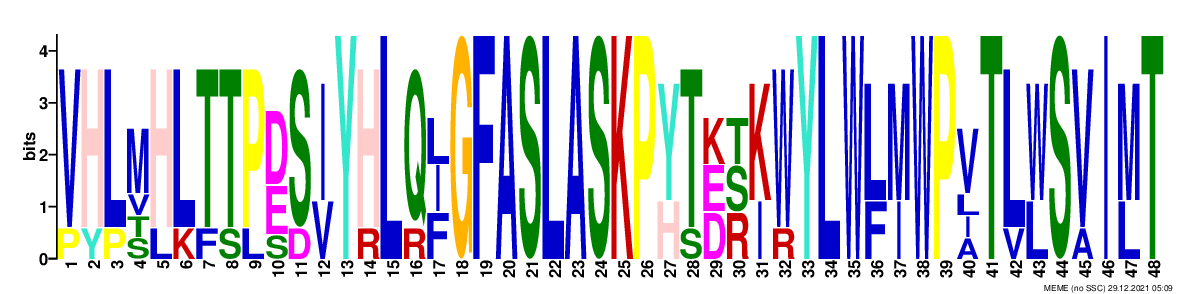VHLMHLTTPDSIYHLQLGFASLASKPYTESKWYLWLMWPVTLWSVIMT | **2.0e-126** |
| **12** | 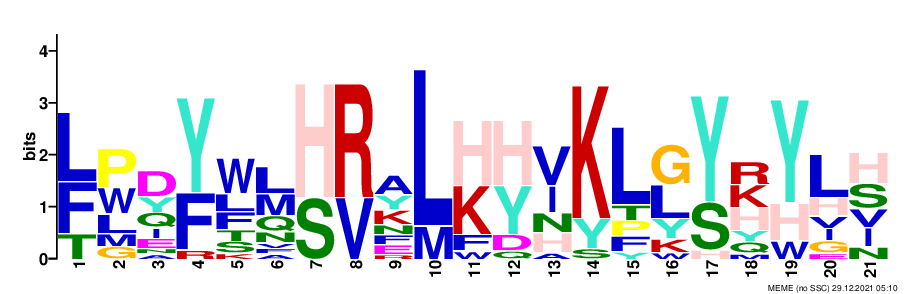LPDYWLHRALHHVKLGYKYLH | **7.3e-080** |
| **13** | 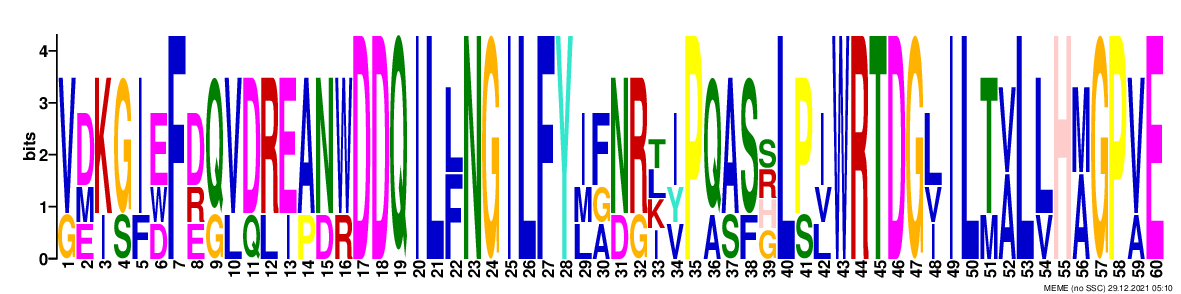VDKGIEFDQVDREANWDDQILFNGILFYIFNRIIPQASGLPIWRTDGJILTVLLHAGPVE | **6.7e-066** |
| **14** | 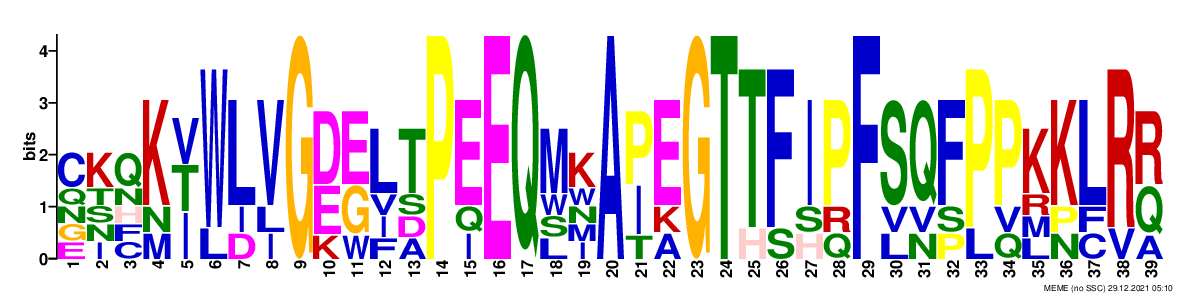CKQKVWLVGDELTPEEQMKAPEGTTFIPFSQFPPKKLRR | **5.8e-055** |
| **15** | 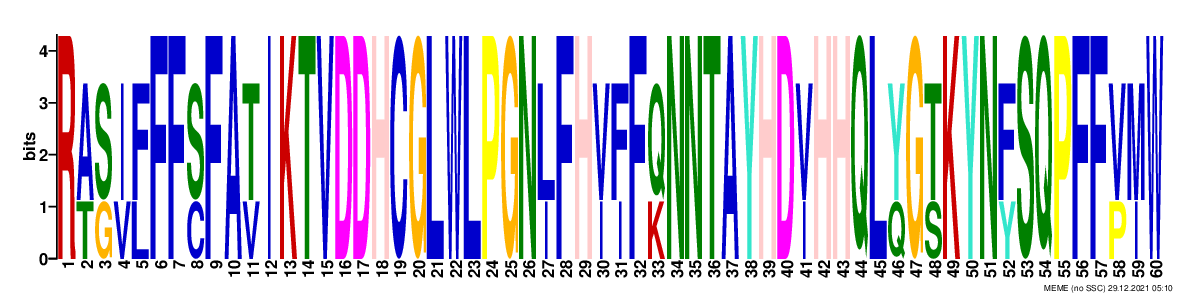RASIFFFSFATIKTVDDHCGLWLPGNLFHVFFQNNTAYHDVHHQLYGTKYNFSQPFFVMW | **3.6e-066** |
