# Supplementary material for: Comprehensive Genome-Wide Identification and Expression Profiling of Eceriferum (CER) Gene Family in Passion Fruit (Passiflora edulis) Under Fusarium kyushuense and Drought Stress Conditions
Source: Front Plant Sci. 2022 Jun 27;13:898307. doi: 10.3389/fpls.2022.898307 (PMC9272567; doi:10.3389/fpls.2022.898307)
Supplement: Supplementary file 1 [file Data_Sheet_1.ZIP › Supplementary Materials/Supplementary Table S6.docx]

| **Supplementary Table S6. Ka/Ks analysis and estimated divergence time for tandem or segmental duplicated *PeCER* genes.** | | | | | | |
| --- | --- | --- | --- | --- | --- | --- |
| **Gene 1** | **Gene 2** | **Ka** | **Ks** | **Ka/Ks** | **Time (mya^)*^** | **Mode of duplication** |
| *PeCER*6 | *PeCER*9 | 0.04 | 0.62 | 0.07 | 16.87 | Tandem |
| *PeCER*8 | *PeCER*27 | 0.04 | 0.76 | 0.05 | 20.78 | Segmental |
| *PeCER*7 | *PeCER*28 | 0.08 | 0.55 | 0.14 | 15.12 | Segmental |
| *PeCER*1 | *PeCER*31 | 0.05 | 0.75 | 0.06 | 20.51 | Segmental |
| *PeCER*13 | *PeCER*14 | 0.07 | 0.14 | 0.47 | 3.86 | Tandem |
| *PeCER*14 | *PeCER*25 | 0.22 | 0.67 | 0.32 | 18.49 | Segmental |
| *PeCER*13 | *PeCER*25 | 0.16 | 0.55 | 0.29 | 15.03 | Segmental |
| *PeCER*20 | *PeCER*29 | 0.03 | 0.04 | 0.68 | 1.12 | Segmental |
| Time = Ks/2x where x is 6.38 10^-9^;  ^*^mya=millions years ago. | | | | | | |
